# Multicenter neonatal databases: Trends in research uses

**DOI:** 10.1186/s13104-016-2336-4

**Published:** 2017-01-13

**Authors:** Liza M. Creel, Sean Gregory, Catherine J. McNeal, Madhava R. Beeram, David R. Krauss

**Affiliations:** 1 Department of Health Management and Systems Science, School of Public Health & Information Sciences, University of Louisville, Louisville, USA; 2 Department of Health Policy and Management, College of Public Health, University of South Florida, 13201 Bruce B Downs Blvd, MDC 56, Tampa, FL 33616 USA; 3 Department of Pediatrics, Morsani College of Medicine, University of South Florida, Tampa, USA; 4 Department of Psychiatry, Morsani College of Medicine, University of South Florida, Tampa, USA; 5 Department of Pediatrics, Baylor Scott & White Health, Temple, USA; 6 Department of Internal Medicine, Baylor Scott & White Health, Temple, USA

**Keywords:** NICU outcomes, Pediatrics outcomes, NICU databases, Prematurity

## Abstract

**Background:**

In the US, approximately 12.7% of all live births are preterm, 8.2% of live births were low birth weight (LBW), and 1.5% are very low birth weight (VLBW). Although technological advances have improved mortality rates among preterm and LBW infants, improving overall rates of prematurity and LBW remains a national priority. Monitoring short- and long-term outcomes is critical for advancing medical treatment and minimizing morbidities associated with prematurity or LBW; however, studying these infants can be challenging. Several large, multi-center neonatal databases have been developed to improve research and quality improvement of treatments for and outcomes of premature and LBW infants. The purpose of this systematic review was to describe three multi-center neonatal databases.

**Methods:**

We conducted a literature search using PubMed and Google Scholar over the period 1990 to August 2014. Studies were included in our review if one of the databases was used as a primary source of data or comparison. Included studies were categorized by year of publication; study design employed, and research focus.

**Results:**

A total of 343 studies published between 1991 and 2014 were included. Studies of premature and LBW infants using these databases have increased over time, and provide evidence for both neonatology and community-based pediatric practice.

**Conclusions:**

Research into treatment and outcomes of premature and LBW infants is expanding, partially due to the availability of large, multicenter databases. The consistency of clinical conditions and neonatal outcomes studied since 1990 demonstrates that there are dedicated research agendas and resources that allow for long-term, and potentially replicable, studies within this population.

**Electronic supplementary material:**

The online version of this article (doi:10.1186/s13104-016-2336-4) contains supplementary material, which is available to authorized users.

## Background

Each year, prematurity and low birth weight (LBW) impacts a small but significant proportion of all live births in the United States. In the United States and internationally, improving outcomes for these infants remains a high priority. Healthy people 2020 includes objectives to reduce LBW and premature births [1]. These objective include targets to reduce overall preterm births from 12.7 to 11.4% as well as specific objectives to reduce both very preterm and late preterm births by 10%, and to reduce the number of LBW (8.2–7.8%), and very LBW (VLBW) infants (1.5–1.4%) [1].

In the United States, overall infant mortality has declined from 100 per 1000 live births in 1900 to 6.05 per 1000 births in 2011 [2] while remaining one of the highest infant mortality rates among industrialized countries [3]. The development of medical and technological interventions has improved the survivability of premature and LBW infants. From 2000 to 2010 the infant mortality rate among preterm infants decreased from 37.88 deaths under age 1 per 1000 live births to 34.22 per 1000 live births, a decrease of almost 10 percent [4]. For infants born under 32 weeks, the mortality rate decreased almost 8% from 2000 to 2010, from 180.95 per 1000 live births to 165.57 per 1000 live births [4]. While overall infant mortality rates have decreased, they are still comparatively high and short- and long-term morbidities associated with prematurity and LBW have persisted [4, 5]. There are a number of potential morbidities that affect nearly every organ system and include conditions such as poor neurodevelopmental outcomes, retinopathy of prematurity, severe intraventricular hemorrhage, hearing loss, bronchopulmonary dysplasia, respiratory distress syndrome, patent ductus arteriosus, necrotizing enterocolitis, and sepsis are associated with LBW and prematurity, and may be complicated by interventions to improve mortality such as ventilation [5–13], transfusions and catheters. Common interventions provided during a typical neonatal intensive care unit (NICUs) hospitalization.

Monitoring both short- and long-term outcomes of infants affected by preterm birth, LBW and/VLBW is critical to advancing scientific and medical knowledge with respect to the development of more effective treatment guidelines, to improve quality of these treatments over time, and to minimize short- and long-term morbidities. Effective research can also inform integrated health care practices where surviving infants are treated through childhood and even into adulthood. However, studying infants affected by prematurity or LBW can be challenging due to small, single-center sample sizes, unknown quality of some administrative data, or limited availability of long-term follow-up data.

To address these challenges, a number of large-scale databases were developed to allow structured study of premature and LBW infants, including but not limited to those admitted to the NICU. In 1997, Wright and Papile [14] summarized existing neonatal databases and their uses. Their review provided detailed descriptions of four neonatal databases: the Kaiser Permanente Neonatal Minimum Data Set (KPNMDS), the Vermont Oxford Network (VON), the Eunice Kennedy Shriver National Institute of Child Health and Human Development Neonatal Research Network (NICHD NRN), and the National Perinatal Information Center (NPIC). Since 1997, there have been tremendous advances in neonatal care that have contributed to declines in infant mortality associated with prematurity or LBW, including the use of high-frequency ventilation and cooling caps. These clinical improvements are accompanied by an increasing number of studies aimed at evaluating neonatal intervention, understanding the progression of disease, and investigating outcomes of those infants affected by prematurity or LBW. The purpose of our current review is to provide an update on three of these databases, and to describe their use in epidemiologic studies, the study of specific clinical conditions associated with prematurity and LBW, and clinical outcomes for those infants.

### Existing databases

Of the four originally described by White and Papile, we are reviewing research progress using three, KPNMDS, NICHD NRN, and VON [14]. The National Perinatal Information Center was not included in our review as their focus is on the perinatal period and not premature or LBW infants. The three databases have varying program goals, funding sources, strategies for data collection, and length of follow-up, but all focus on improving medical knowledge about and the quality of care provided to premature, LBW, and NICU admitted infants.

The Kaiser Permanente Neonatal Minimum Data Set (KPNMDS) originated in 1992 and is internally funded through the Kaiser Permanente (KP) system [15]. The KPNMDS was developed to obtain reliable data about the NICU admission, and to support research and quality improvement efforts. The database includes both inborn and outborn admissions to at least six KP NICUs in Northern California, although the total number of NICUs participating in KPNMDS has increased since Wright and Papile described the database in 1997. The KPNMDS includes data on the full NICU admission, and some prospective studies using KPNMDS data extend follow-up for months or years after discharge from the NICU. The primary criterion for inclusion in the database is NICU admission, not a specific birth weight or gestational age. KPNMDS supports both retrospective and prospective studies. Although KPNMDS focuses on NICU admissions, it is relevant to our study as many premature and LBW infants are treated in NICUs.

The Vermont Oxford Network (VON) originated in 1989 and seeks to “improve the quality and safety of medical care for newborn infants and their families through a coordinated program of research, education, and quality improvement projects” [16]. VON maintains two international databases, the very low birth weight database and the expanded database, with a total of over two million infant cases [16]. The very low birth weight database includes inborn and outborn (if admitted within 28 days of birth) infants with birth weights below 1500 g or gestational ages between 22 weeks 0 days and 29 weeks 6 days [17]. The Expanded Database includes all infants from the very low birth weight database as well as infants born at more than 1500 g and admitted to a NICU at a participating center, or “who die at any location in the center within 28 days of birth without first having gone home” [17]. In 2012, VON reported 369 centers reporting data on 153,093 infants into the expanded database, and 909 centers reporting data on 60,007 infants into the VLBW database [17]. VON members pay an annual membership fee and are eligible to use the data for studies, given strict adherence to data use guidelines set forth by VON leadership [18]. In general, VON includes infant data through discharge, death, or one year of age although some prospective studies using VON have longer follow-up periods. VON supports both retrospective and prospective studies. NICUs may apply to participate in VON using a membership application and must pay a membership fee.

The Eunice Kennedy Shriver National Institute of Child Health and Human Development (NICHD) Neonatal Research Network (NRN) began in 1986 and includes a registry to house data from multiple clinical trials funded through NICHD. NICHD supports the NRN financially. Originally, the NRN registry included data for inborn and outborn infants having a birth weight between 401 and 1500 g [14]. Since 2008, the database has included only inborn infants with a gestational age between 22 0/7 to 28 6/7 weeks and/or a birth weight between 401 and 1000 g [19]. It also includes follow-up data at 18–26 months, depending on the year of study and if the participating study site(s) assessed outcomes at such age as part of their research protocol [14, 19]. As of August 1, 2014, the NRN website listed 20 participating sites [20]. NICHD NRN supports both retrospective and prospective studies, specifically clinical intervention and epidemiologic studies funded through NICHD. Participation in the NICHD NRN requires funding through NICHD, which is typically provided through a competitive grant process.

All three databases use standardized forms and definitions for data submission by participating sites. In general, data use is open to participating sites contributing data to the database as long as database-specific requirements are met.

These databases have continued to expand and become more widely used since they were first reviewed in 1997. In the sections below we provide a summary of how the databases are being used to advance scientific and clinical knowledge about the epidemiology of prematurity and LBW and the clinical treatment of those infants. We focus specifically on database use in studies published in the peer-reviewed literature and provide aggregate information on study designs and areas of clinical focus. Our purpose is to offer clinical and health services researchers insight into how research on preterm and LBW infants has evolved, and to offer strengths and opportunities for continued research using these and other databases.

## Methods

We conducted a literature search using PubMed and Google Scholar over the time period from January 1990 to August 15, 2014. Search terms included official names for each of the databases and their abbreviations, if applicable. For example, the Kaiser Permanente Neonatal Minimum Data Set was searched for using the full name as well as “KP Neonatal Minimum Data Set” and “Kaiser Permanente Neonatal MDS.” None of the databases were searched for simultaneously, although several studies were returned during separate database searches.

Article titles and abstracts were reviewed for inclusion in our study. Initial inclusion criteria only required that the article include the name of one of the three databases and there was some evidence from the abstract that the study used or participated in the database network. Initial results were compared with publication lists maintained by the database managers. Both VON and NICHD NRN maintained such lists, which were last reviewed on August 15, 2014. In both cases, additional studies were added into our review.

After title and abstract review, all articles were read to determine if the study used the database of interest as a data source for measuring the research question. The database could be used as a primary source of data or as a source of comparison or benchmark data. If either condition was true, the article was included in our study. Exclusion criteria included the following: descriptive articles summarizing database use or methodology, articles using similar but tangential databases such as the VON Encephalopathy Registry or Moderately Premature Infant Project database, articles referencing only definitions or tools (e.g. SNAP-II and SNAPPE-II) derived from or used within one of the databases, studies evaluating instrumentation or measurement technology, studies evaluating quality improvement processes implemented at study sites participating in one of the database networks, review articles or meta-analyses, and non-English articles.

Studies were categorized as having either a retrospective or prospective study design. Retrospective studies were further sorted into categories based on use of the database as a primary data source or using it or its published findings as a comparison or benchmark for another study. A primary and secondary clinical focus area was also assigned to each individual study, in an effort to determine study trends. To capture the overall clinical or outcome focus of the study, clinical focus area categories were applied first by the primary investigator, then reviewed by three other investigators for consistency.

No written informed consent for participation in the study was obtained from participants or, where participants are children, a parent or guardian, as this study was a literature review and synthesis and contained to subjects.

## Results

**Fig. 1:**
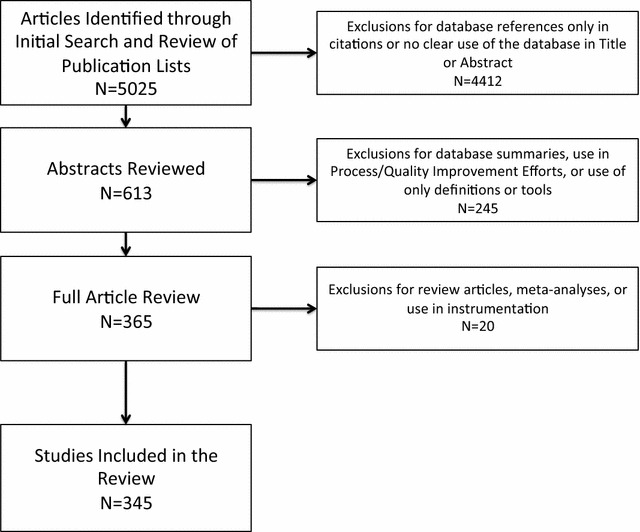
Flow chart of article abstraction process

A final total of 343 studies published between 1990 and 2014 were included in the review. Figure 1 summarizes the abstraction process and the final number of studies included across databases.

**Fig. 2:**
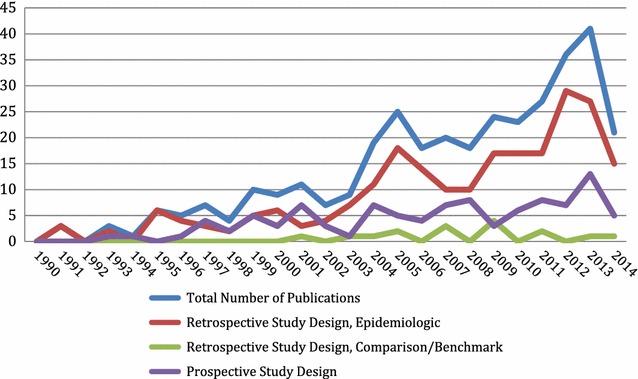
Publications, by year and type of study design

The total number of publications using the databases has increased, from three in 1991 to 41 in 2013 (the last full calendar year included in our review). Both prospective and retrospective studies have also increased, with retrospective studies comprising more of the total number of studies in most years. Around 2005, there was a slight decline in the number of studies published, but publications began to increase again after 2006. Figure 2 summarizes the year-by-year results.

Seventy-one percent (71%) of the studies used a retrospective study design and these studies tended to have an epidemiologic focus. Among retrospective studies, the database was sometimes used as a comparison group or benchmark for a single-center study. For example, Pietz, Achanti, Lilien, Stepka, and Mehta studied the incidence of necrotizing enterocolitis (NEC) in a single NICU over the course of 20 years. Their study looked specifically at the incidence of bowel perforation and NEC among a population of infants that were unlikely to have been treated with indomethacin, a nonsteroidal anti-inflammatory drug that can be used to treat very premature infants. Use of indomethacin in this particular NICU was discouraged and the authors emphasize the need to compare results to other centers that may use indomethacin more frequently. The study authors compared results from their NICU to overall results from VON, which likely included infants treated in NICUs employing more typical practice (for the time) of using indomethacin, and tested for differences in rates of NEC [21]. Alternatively, retrospective studies also used the databases to study population health research questions. For example, Stoll, et al. [5] utilized the NICHD Neonatal Research Network database to retrospectively examine trends in morbidity and mortality among LBW infants. Smith et al. [22], used the KPNMDS to study temporal trends in bronchopulmonary dysplasia rates over eight years.

Approximately 31% of studies utilized a prospective study design where the database was used as a sampling frame, or was used to house study data and answer a specific clinical research question. In their study of neurodevelopmental outcomes among extremely LBW infants (i.e., <2000 g), Mercier et al. [23], used the VON database as a sampling frame from which infants were identified for follow-up assessments. Lorch, Srinivasan, and Escobar published a study on the epidemiology of apnea and brachycardia in premature infants, which used the KPNMDS as a primary data source throughout the infants’ admission to the NICU [24].

Studies focused on a variety of clinical conditions, interventions, and outcomes, with just over 70% of studies concentrating on ten categories (summarized in Table 1). The top ten areas of research focus were respiratory treatments/outcomes; neurodevelopmental, growth, or language outcomes; outcomes of very LBW or extremely LBW; encephalopathy; neonatal infections; intestinal disease; sepsis; antenatal corticosteriod treatment; retinopathy of prematurity, and hyperbilirubinemia. The remaining 30% of studies focus on other specific conditions and interventions and account for a large amount of diversity in study focus areas. Approximately 10% of the studies were in a category alone, leaving 90% in categories with two or more studies.

**Table 1:** Primary clinical areas of focus for studies using multicenter NICU databases

Primary clinical focus area	Count	Percent
Respiratory treatments & outcomes	67	19.53
Neurodevelopmental, growth, or language outcomes	45	13.12
Outcomes of VLBW/ELBW	39	11.37
Encephalopathy	24	7.00
Neonatal infections	18	5.25
Intestinal disease	15	4.37
Sepsis	14	4.08
Antenatal corticosteriod treatment	10	2.92
Retinopathy of prematurity	8	2.33
Hyperbilirubinemia	8	2.33
Other	95	27.70
Total	343	100

Among those studies in the top ten categories, some were given a secondary clinical focus area to further describe the research. This occurred frequently in the broader categories looking at outcomes. For example, studies focusing on respiratory treatment and outcomes may have specific research questions related to use of surfactant or comparing ventilation strategies. Studies of neurodevelopmental outcomes tended to have secondary clinical foci on specific clinical conditions such as NEC, intraventricular hemorrhage, or hyperbilirubinemia. Other top categories, such as intestinal disease, had fewer secondary categories due, presumably, to the focus of the topic area.

**Fig. 3:**
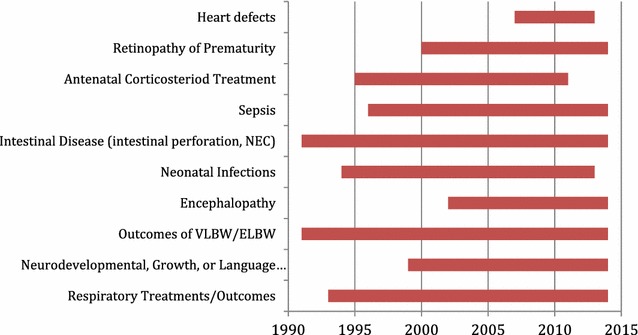
Topics of study by year

Studies in each category also varied in terms of the range of time in which they were published. The earliest published studies focus on intestinal disease and overall outcomes of VLBW and ELBW infants, and these tend to continue through the duration of time included in our review. Alternatively, studies of heart defects and encephalopathy using one of the three databases were not published until after 2000. Figure 3 below describes the range of years across which studies in the top categories were published.

## Conclusions

Although birth outcomes such as prematurity and LBW may be relatively rare, infants with these outcomes and related conditions are more likely now than ever to survive their birth admission and receive community-based care in infancy and childhood. Often this care requires treatment of morbidities or chronic conditions associated with birth status or treatment thereafter, which motivates researchers to study short- and long-term outcomes that inform the practice of neonatology and pediatrics.

Three large databases focusing on premature, LBW, and/or very acutely ill neonates are available to researchers seeking to understand and improve birth and long-term outcomes for those infants. To date, an increasing number of studies using the three neonatal research databases have been published in the literature and these studies use both prospective and retrospective research designs.

The studies include research in clinical areas important to advancing neonatal and pediatric medicine. Ten studies included in this review are part of the body of research on antenatal steroid use in mothers at risk of preterm delivery, and have contributed to the body of research demonstrating both the risks and benefits of antenatal steroid use. Over 20 studies focus on encephalopathy, a condition affecting moderately premature infants. This is an important enough issue in medicine that an entirely separate registry was developed by the VON to allow for quality improvement and research efforts specific to encephalopathy.

The databases included in this review (and others) offer several advantages for researchers. First, each of the databases includes a large number of infants allowing for larger sample sizes, especially when studying rarer conditions such as heart defects. Second, the databases include infants born or treated at multiple centers from diverse geographies, improving the likelihood of obtaining generalizable results in epidemiologic studies. Finally, the databases each have significant administrative guidelines and support, which provides researchers with valid and reliable data.

While a large proportion of studies focus on outcomes, there are variations in how long these outcomes are monitored within each database. The length of follow-up within each database varies, with many studies following infants through discharge from the NICU or hospital, and others, especially those with prospective data collection, follow infants into childhood. Nevertheless, the ability for consistent durations of long-term follow-up is currently limited. Researchers seeking to study disease epidemiology and long-term outcomes may find opportunities with single center databases with smaller sample sizes. Single-center retrospective databases may offer data that are easier to obtain, administratively, and may be available for many years on each infant.

This review of studies utilizing data from existing neonatal databases expands on the work of Wright and Papile [14] and provides new information about how research on premature and LBW infants is evolving. Our review is limited by a very focused search strategy that used the database names and abbreviations as the only search terms, which may have caused us to miss some studies that used the databases but did not reference the data source in the same way we searched. Even so, our search yielded over 345 studies that were ultimately included in the review and we believe that this provides adequate power to show trends in this research area (Additional file 1).

Research into treatment and outcomes of premature and LBW infants is expanding, partially due to the availability of large, multicenter databases. The consistency of clinical conditions and neonatal outcomes studied since 1990 demonstrates that there are dedicated research agendas and resources that allow for long-term, and potentially replicable, studies within this population. Alternatively, the diversity of research topics and outcomes establishes an environment in which researchers can study new and innovative interventions or even some of the more rare conditions for which premature and LBW infants are at risk. These trends in neonatal research, specifically research focused on premature and LBW infants, offer a strong foundation for future research efforts to inform neonatology, pediatric and perhaps even adult medicine with the remarkable improvements in survivability and improved long-term outcomes for these medically fragile infants.

## Additional file

**Additional file 1.** Additional table and references.

## Abbreviations

LBW: low birth weight
VLBW: very low birth weight
ELBW: extremely low birth weight
NICU: neonatal intensive care unit
KPNMDS: Kaiser permanente neonatal minimum data set
VON: Vermont Oxford Network
NICHD NRN: Eunice Kennedy Shriver National Institute of Child Health and Human Development Neonatal Research Network
NPIC: National Perinatal Information Center
NEC: necrotizing enterocolitis
