# Supplementary material for: Multicenter neonatal databases: Trends in research uses
Source: BMC Res Notes. 2017 Jan 13;10:42. doi: 10.1186/s13104-016-2336-4 (PMC5237182; doi:10.1186/s13104-016-2336-4)
Supplement: Supplementary file 1 — Additional file 1. Additional table and references. [file 13104_2016_2336_MOESM1_ESM.docx]

**Table 1: Primary Clinical Areas of Focus for Studies Using Multicenter NICU Databases**

| **Primary Clinical Focus Area** | **Count** | **Percent (%)** | **Citations** |
| --- | --- | --- | --- |
| Respiratory Treatments & Outcomes | 67 | 19.53% | ^1-66^ |
| Neurodevelopmental, Growth, or Language Outcomes | 45 | 13.12% | ^67-111^ |
| Outcomes of VLBW/ELBW | 39 | 11.37% | ^112-150^ |
| Encephalopathy | 24 | 7.00% | ^151-174^ |
| Neonatal Infections | 18 | 5.25% | ^175-192^ |
| Intestinal Disease | 15 | 4.37% | ^193-207^ |
| Sepsis | 14 | 4.08% | ^208-221^ |
| Antenatal Corticosteriod Treatment | 10 | 2.92% | ^222-231^ |
| Retinopathy of Prematurity | 8 | 2.33% | ^232-239^ |
| Hyperbilirubinemia | 8 | 2.33% | ^240-247^ |
| Other | 95 | 27.70% | ^248-343^ |
| **Total** | **343** | **100%** |  |

**References**

1. Escobar GJ, Gebretsadik T, Carroll K, Li SX, Walsh EM, Wu P, et al. Adherence to Immunoprophylaxis Regimens for Respiratory Syncytial Virus Infection in Insured and Medicaid Populations. *Journal of the Pediatric Infectious Diseases Society*. 2013;2(3):205-214.

2. Kuzniewicz M, Draper D, Escobar GJ. Incorporation of physiological trend and interaction effects in neonatal severity of illness scores: an experiment using a variant of the Richardson score. *Intensive care medicine*. 2007;33(9):1602-1608.

3. Wilson A, Gardner MN, Armstrong MA, Folck BF, Escobar GJ. Neonatal assisted ventilation: predictors, frequency, and duration in a mature managed care organization. *Pediatrics*. 2000;105(4):822-830.

4. Smith VC, Zupancic JA, McCormick MC, Croen LA, Greene J, Escobar GJ, et al. Rehospitalization in the first year of life among infants with bronchopulmonary dysplasia. *The Journal of pediatrics*. 2004;144(6):799-803.

5. Escobar GJ, Ragins A, Li SX, Prager L, Masaquel AS, Kipnis P. Recurrent wheezing in the third year of life among children born at 32 weeks' gestation or later: relationship to laboratory-confirmed, medically attended infection with respiratory syncytial virus during the first year of life. *Archives of pediatrics & adolescent medicine*. 2010;164(10):915-922.

6. Escobar GJ, Shaheen SM, Breed EM, Botas C, Greene JD, Yoshida CK, et al. Richardson score predicts short-term adverse respiratory outcomes in newborns≥ 34 weeks gestation. *The Journal of pediatrics*. 2004;145(6):754-760.

7. Lorch SA, Wade KC, Bakewell-Sachs S, Medoff-Cooper B, Escobar GJ, Silber JH. Racial differences in the use of respiratory medications in premature infants after discharge from the neonatal intensive care unit. *The Journal of pediatrics*. 2007;151(6):604-610. e601.

8. Anadkat J, Kuzniewicz M, Chaudhari B, Cole F, Hamvas A. Increased risk for respiratory distress among white, male, late preterm and term infants. *Journal of Perinatology*. 2012;32(10):780-785.

9. Smith VC, Zupancic JA, McCormick MC, Croen LA, Greene J, Escobar GJ, et al. Trends in severe bronchopulmonary dysplasia rates between 1994 and 2002. *The Journal of pediatrics*. 2005;146(4):469-473.

10. Ambalavanan N, Carlo WA, D'Angio CT, McDonald SA, Das A, Schendel D, et al. Cytokines associated with bronchopulmonary dysplasia or death in extremely low birth weight infants. *Pediatrics*. 2009;123(4):1132-1141.

11. Ehrenkranz RA, Walsh MC, Vohr BR, Jobe AH, Wright LL, Fanaroff AA, et al. Validation of the National Institutes of Health consensus definition of bronchopulmonary dysplasia. *Pediatrics*. 2005;116(6):1353-1360.

12. Kennedy KA, Stoll BJ, Ehrenkranz RA, Oh W, Wright LL, Stevenson DK, et al. Vitamin A to prevent bronchopulmonary dysplasia in very-low-birth-weight infants: has the dose been too low? The NICHD Neonatal Research Network. *Early human development*. 1997;49(1):19-31.

13. Laughon MM, Langer JC, Bose CL, Smith PB, Ambalavanan N, Kennedy KA, et al. Prediction of bronchopulmonary dysplasia by postnatal age in extremely premature infants. *American journal of respiratory and critical care medicine*. 2011;183(12):1715-1722.

14. Natarajan G, Pappas A, Shankaran S, Kendrick DE, Das A, Higgins RD, et al. Outcomes of extremely low birth weight infants with bronchopulmonary dysplasia: impact of the physiologic definition. *Early human development*. 2012;88(7):509-515.

15. Oh W, Poindexter BB, Perritt R, Lemons JA, Bauer CR, Ehrenkranz RA, et al. Association between fluid intake and weight loss during the first ten days of life and risk of bronchopulmonary dysplasia in extremely low birth weight infants. *The Journal of pediatrics*. 2005;147(6):786-790.

16. Walsh MC, Yao Q, Gettner P, Hale E, Collins M, Hensman A, et al. Impact of a physiologic definition on bronchopulmonary dysplasia rates. *Pediatrics*. 2004;114(5):1305-1311.

17. Ambalavanan N, Walsh M, Bobashev G, Das A, Levine B, Carlo WA, et al. Intercenter differences in bronchopulmonary dysplasia or death among very low birth weight infants. *Pediatrics*. 2011;127(1):e106-116.

18. Fernandez E, Watterberg KL, Faix RG, Yoder BA, Walsh MC, Lacy CB, et al. Incidence, Management, and Outcomes of Cardiovascular Insufficiency in Critically Ill Term and Late Preterm Newborn Infants. *American journal of perinatology*. 2014.

19. Leitch CA, Ahlrichs J, Karn C, Denne SC. Energy expenditure and energy intake during dexamethasone therapy for chronic lung disease. *Pediatr Res*. 1999;46(1):109-113.

20. Finer NN, Carlo WA, Duara S, Fanaroff AA, Donovan EF, Wright LL, et al. Delivery room continuous positive airway pressure/positive end-expiratory pressure in extremely low birth weight infants: a feasibility trial. *Pediatrics*. 2004;114(3):651-657.

21. Vaucher YE, Peralta-Carcelen M, Finer NN, Carlo WA, Gantz MG, Walsh MC, et al. Neurodevelopmental outcomes in the early CPAP and pulse oximetry trial. *N Engl J Med*. 2012;367(26):2495-2504.

22. Papile LA, Tyson JE, Stoll BJ, Wright LL, Donovan EF, Bauer CR, et al. A multicenter trial of two dexamethasone regimens in ventilator-dependent premature infants. *N Engl J Med*. 1998;338(16):1112-1118.

23. Stark AR, Carlo WA, Tyson JE, Papile LA, Wright LL, Shankaran S, et al. Adverse effects of early dexamethasone in extremely-low-birth-weight infants. National Institute of Child Health and Human Development Neonatal Research Network. *N Engl J Med*. 2001;344(2):95-101.

24. Stark AR, Carlo WA, Vohr BR, Papile LA, Saha S, Bauer CR, et al. Death or neurodevelopmental impairment at 18 to 22 months corrected age in a randomized trial of early dexamethasone to prevent death or chronic lung disease in extremely low birth weight infants. *The Journal of pediatrics*. 2014;164(1):34-39.e32.

25. LeVan JM, Wyckoff MH, Ahn C, Heyne R, Sanchez PJ, Chalak L, et al. Change in care among nonenrolled patients during and after a randomized trial. *Pediatrics*. 2013;132(4):e960-970.

26. Konduri GG, Solimano A, Sokol GM, Singer J, Ehrenkranz RA, Singhal N, et al. A randomized trial of early versus standard inhaled nitric oxide therapy in term and near-term newborn infants with hypoxic respiratory failure. *Pediatrics*. 2004;113(3 Pt 1):559-564.

27. Konduri GG, Vohr B, Robertson C, Sokol GM, Solimano A, Singer J, et al. Early inhaled nitric oxide therapy for term and near-term newborn infants with hypoxic respiratory failure: neurodevelopmental follow-up. *The Journal of pediatrics*. 2007;150(3):235-240, 240.e231.

28. The Neonatal Inhaled Nitric Oxide Study Group. Inhaled nitric oxide in full-term and nearly full-term infants with hypoxic respiratory failure. *N Engl J Med*. 1997;336(9):597-604.

29. Sokol GM, Fineberg NS, Wright LL, Ehrenkranz RA. Changes in arterial oxygen tension when weaning neonates from inhaled nitric oxide. *Pediatric pulmonology*. 2001;32(1):14-19.

30. Van Meurs KP, Hintz SR, Ehrenkranz RA, Lemons JA, Ball MB, Poole WK, et al. Inhaled nitric oxide in infants >1500 g and <34 weeks gestation with severe respiratory failure. *Journal of perinatology : official journal of the California Perinatal Association*. 2007;27(6):347-352.

31. Van Meurs KP, Wright LL, Ehrenkranz RA, Lemons JA, Ball MB, Poole WK, et al. Inhaled nitric oxide for premature infants with severe respiratory failure. *N Engl J Med*. 2005;353(1):13-22.

32. Konduri GG, Sokol GM, Van Meurs KP, Singer J, Ambalavanan N, Lee T, et al. Impact of early surfactant and inhaled nitric oxide therapies on outcomes in term/late preterm neonates with moderate hypoxic respiratory failure. *Journal of perinatology : official journal of the California Perinatal Association*. 2013;33(12):944-949.

33. Laptook AR, Salhab W, Allen J, Saha S, Walsh M. Pulse oximetry in very low birth weight infants: can oxygen saturation be maintained in the desired range? *Journal of perinatology : official journal of the California Perinatal Association*. 2006;26(6):337-341.

34. Truog WE, Nelin LD, Das A, Kendrick DE, Bell EF, Carlo WA, et al. Inhaled nitric oxide usage in preterm infants in the NICHD neonatal research network: inter-site variation and propensity evaluation. *Journal of perinatology : official journal of the California Perinatal Association*. 2014.

35. Walsh MC, Morris BH, Wrage LA, Vohr BR, Poole WK, Tyson JE, et al. Extremely low birthweight neonates with protracted ventilation: mortality and 18-month neurodevelopmental outcomes. *The Journal of pediatrics*. 2005;146(6):798-804.

36. Wang K, Difiore JM, Martin RJ, Rosen CL, Hibbs AM. Markers for severity of illness associated with decreased snoring in toddlers born ELGA. *Acta paediatrica (Oslo, Norway : 1992)*. 2013;102(1):e39-43.

37. Chock VY, Van Meurs KP, Hintz SR, Ehrenkranz RA, Lemons JA, Kendrick DE, et al. Inhaled nitric oxide for preterm premature rupture of membranes, oligohydramnios, and pulmonary hypoplasia. *American journal of perinatology*. 2009;26(4):317-322.

38. Walsh M, Laptook A, Kazzi SN, Engle WA, Yao Q, Rasmussen M, et al. A cluster-randomized trial of benchmarking and multimodal quality improvement to improve rates of survival free of bronchopulmonary dysplasia for infants with birth weights of less than 1250 grams. *Pediatrics*. 2007;119(5):876-890.

39. Thomas CW, Meinzen-Derr J, Hoath SB, Narendran V. Neurodevelopmental outcomes of extremely low birth weight infants ventilated with continuous positive airway pressure vs. mechanical ventilation. *Indian journal of pediatrics*. 2012;79(2):218-223.

40. Finer NN, Carlo WA, Walsh MC, Rich W, Gantz MG, Laptook AR, et al. Early CPAP versus surfactant in extremely preterm infants. *N Engl J Med*. 2010;362(21):1970-1979.

41. Di Fiore JM, Walsh M, Wrage L, Rich W, Finer N, Carlo WA, et al. Low oxygen saturation target range is associated with increased incidence of intermittent hypoxemia. *The Journal of pediatrics*. 2012;161(6):1047-1052.

42. St John EB, Carlo WA. Respiratory distress syndrome in VLBW infants: changes in management and outcomes observed by the NICHD Neonatal Research Network. *Seminars in perinatology*. 2003;27(4):288-292.

43. Ambalavanan N, Van Meurs KP, Perritt R, Carlo WA, Ehrenkranz RA, Stevenson DK, et al. Predictors of death or bronchopulmonary dysplasia in preterm infants with respiratory failure. *Journal of perinatology : official journal of the California Perinatal Association*. 2008;28(6):420-426.

44. Horbar JD, Wright EC, Onstad L. Decreasing mortality associated with the introduction of surfactant therapy: an observational study of neonates weighing 601 to 1300 grams at birth. The Members of the National Institute of Child Health and Human Development Neonatal Research Network. *Pediatrics*. 1993;92(2):191-196.

45. Horbar JD, Wright LL, Soll RF, Wright EC, Fanaroff AA, Korones SB, et al. A multicenter randomized trial comparing two surfactants for the treatment of neonatal respiratory distress syndrome. National Institute of Child Health and Human Development Neonatal Research Network. *The Journal of pediatrics*. 1993;123(5):757-766.

46. DeMauro SB, D'Agostino JA, Bann C, Bernbaum J, Gerdes M, Bell EF, et al. Developmental outcomes of very preterm infants with tracheostomies. *The Journal of pediatrics*. 2014;164(6):1303-1310.e1302.

47. Carlo WA, Stark AR, Wright LL, Tyson JE, Papile LA, Shankaran S, et al. Minimal ventilation to prevent bronchopulmonary dysplasia in extremely-low-birth-weight infants. *The Journal of pediatrics*. 2002;141(3):370-374.

48. Bhandari V, Finer NN, Ehrenkranz RA, Saha S, Das A, Walsh MC, et al. Synchronized nasal intermittent positive-pressure ventilation and neonatal outcomes. *Pediatrics*. 2009;124(2):517-526.

49. Dunn MS, Kaempf J, de Klerk A, de Klerk R, Reilly M, Howard D, et al. Randomized trial comparing 3 approaches to the initial respiratory management of preterm neonates. *Pediatrics*. 2011;128(5):e1069-1076.

50. Hentschel J, Berger TM, Tschopp A, Muller M, Adams M, Bucher HU. Population-based study of bronchopulmonary dysplasia in very low birth weight infants in Switzerland. *Eur J Pediatr*. 2005;164(5):292-297.

51. Mandy G, Malkar M, Welty SE, Brown R, Shepherd E, Gardner W, et al. Tracheostomy placement in infants with bronchopulmonary dysplasia: safety and outcomes. *Pediatric pulmonology*. 2013;48(3):245-249.

52. Payne NR, LaCorte M, Karna P, Chen S, Finkelstein M, Goldsmith JP, et al. Reduction of bronchopulmonary dysplasia after participation in the Breathsavers Group of the Vermont Oxford Network Neonatal Intensive Care Quality Improvement Collaborative. *Pediatrics*. 2006;118 Suppl 2:S73-77.

53. Pinheiro JM, Boynton S, Furdon SA, Dugan R, Reu-Donlon C. Use of chemical warming packs during delivery room resuscitation is associated with decreased rates of hypothermia in very low-birth-weight neonates. *Advances in neonatal care : official journal of the National Association of Neonatal Nurses*. 2011;11(5):357-362.

54. Group TVONSS. Early postnatal dexamethasone therapy for the prevention of chronic lung disease. *Pediatrics*. 2001;108(3):741-748.

55. Kirchner L, Weninger M, Unterasinger L, Birnbacher R, Hayde M, Krepler R, et al. Is the use of early nasal CPAP associated with lower rates of chronic lung disease and retinopathy of prematurity? Nine years of experience with the Vermont Oxford Neonatal Network. *Journal of perinatal medicine*. 2005;33(1):60-66.

56. Rudiger M, Ifflander S, Reichert J, Batzel C, Reiter G, Wauer RR. Which information will be given to parents of preterm infants--a comparison of estimates and local data. *Journal of perinatal medicine*. 2007;35(5):436-442.

57. Klebermass-Schrehof K, Wald M, Schwindt J, Grill A, Prusa AR, Haiden N, et al. Less invasive surfactant administration in extremely preterm infants: impact on mortality and morbidity. *Neonatology*. 2013;103(4):252-258.

58. Payne NR, Finkelstein MJ, Liu M, Kaempf JW, Sharek PJ, Olsen S. NICU practices and outcomes associated with 9 years of quality improvement collaboratives. *Pediatrics*. 2010;125(3):437-446.

59. Horbar JD, Carpenter JH, Buzas J, Soll RF, Suresh G, Bracken MB, et al. Collaborative quality improvement to promote evidence based surfactant for preterm infants: a cluster randomised trial. *BMJ (Clinical research ed)*. 2004;329(7473):1004.

60. Horbar JD, Carpenter JH, Buzas J, Soll RF, Suresh G, Bracken MB, et al. Timing of initial surfactant treatment for infants 23 to 29 weeks' gestation: is routine practice evidence based? *Pediatrics*. 2004;113(6):1593-1602.

61. Malkar MB, Gardner WP, Mandy GT, Stenger MR, Nelin LD, Shepherd EG, et al. Respiratory severity score on day of life 30 is predictive of mortality and the length of mechanical ventilation in premature infants with protracted ventilation. *Pediatric pulmonology*. 2014.

62. Vermont-Oxford Neonatal Network. A multicenter, randomized trial comparing synthetic surfactant with modified bovine surfactant extract in the treatment of neonatal respiratory distress syndrome. *Pediatrics*. 1996;97(1):1-6.

63. Schmidt B, Asztalos EV, Roberts RS, Robertson CM, Sauve RS, Whitfield MF. Impact of bronchopulmonary dysplasia, brain injury, and severe retinopathy on the outcome of extremely low-birth-weight infants at 18 months: results from the trial of indomethacin prophylaxis in preterms. *Jama*. 2003;289(9):1124-1129.

64. Walsh MC, Yao Q, Horbar JD, Carpenter JH, Lee SK, Ohlsson A. Changes in the use of postnatal steroids for bronchopulmonary dysplasia in 3 large neonatal networks. *Pediatrics*. 2006;118(5):e1328-1335.

65. Wilson-Costello D, Walsh MC, Langer JC, Guillet R, Laptook AR, Stoll BJ, et al. Impact of postnatal corticosteroid use on neurodevelopment at 18 to 22 months' adjusted age: effects of dose, timing, and risk of bronchopulmonary dysplasia in extremely low birth weight infants. *Pediatrics*. 2009;123(3):e430-437.

66. Kuppala VS, Meinzen-Derr J, Morrow AL, Schibler KR. Prolonged initial empirical antibiotic treatment is associated with adverse outcomes in premature infants. *The Journal of pediatrics*. 2011;159(5):720-725.

67. Oh W, Stevenson DK, Tyson JE, Morris BH, Ahlfors CE, Bender GJ, et al. Influence of clinical status on the association between plasma total and unbound bilirubin and death or adverse neurodevelopmental outcomes in extremely low birth weight infants. *Acta paediatrica (Oslo, Norway : 1992)*. 2010;99(5):673-678.

68. Oh W, Tyson JE, Fanaroff AA, Vohr BR, Perritt R, Stoll BJ, et al. Association between peak serum bilirubin and neurodevelopmental outcomes in extremely low birth weight infants. *Pediatrics*. 2003;112(4):773-779.

69. Carlo WA, McDonald SA, Tyson JE, Stoll BJ, Ehrenkranz RA, Shankaran S, et al. Cytokines and neurodevelopmental outcomes in extremely low birth weight infants. *The Journal of pediatrics*. 2011;159(6):919-925.e913.

70. Vohr BR, Wright LL, Poole WK, McDonald SA. Neurodevelopmental outcomes of extremely low birth weight infants <32 weeks' gestation between 1993 and 1998. *Pediatrics*. 2005;116(3):635-643.

71. Duncan AF, Watterberg KL, Nolen TL, Vohr BR, Adams-Chapman I, Das A, et al. Effect of ethnicity and race on cognitive and language testing at age 18-22 months in extremely preterm infants. *The Journal of pediatrics*. 2012;160(6):966-971.e962.

72. Walden RV, Taylor SC, Hansen NI, Poole WK, Stoll BJ, Abuelo D, et al. Major congenital anomalies place extremely low birth weight infants at higher risk for poor growth and developmental outcomes. *Pediatrics*. 2007;120(6):e1512-1519.

73. Lowe J, Woodward B, Papile LA. Emotional regulation and its impact on development in extremely low birth weight infants. *Journal of developmental and behavioral pediatrics : JDBP*. 2005;26(3):209-213.

74. Lowe JR, Duncan AF, Bann CM, Fuller J, Hintz SR, Das A, et al. Early working memory as a racially and ethnically neutral measure of outcome in extremely preterm children at 18-22 months. *Early human development*. 2013;89(12):1055-1061.

75. Lowe JR, Nolen TL, Vohr B, Adams-Chapman I, Duncan AF, Watterberg K. Effect of primary language on developmental testing in children born extremely preterm. *Acta paediatrica (Oslo, Norway : 1992)*. 2013;102(9):896-900.

76. Ohls RK, Ehrenkranz RA, Das A, Dusick AM, Yolton K, Romano E, et al. Neurodevelopmental outcome and growth at 18 to 22 months' corrected age in extremely low birth weight infants treated with early erythropoietin and iron. *Pediatrics*. 2004;114(5):1287-1291.

77. Adams-Chapman I, Bann CM, Vaucher YE, Stoll BJ. Association between feeding difficulties and language delay in preterm infants using Bayley Scales of Infant Development-Third Edition. *The Journal of pediatrics*. 2013;163(3):680-685.e681-683.

78. Ehrenkranz RA, Younes N, Lemons JA, Fanaroff AA, Donovan EF, Wright LL, et al. Longitudinal growth of hospitalized very low birth weight infants. *Pediatrics*. 1999;104(2 Pt 1):280-289.

79. Ehrenkranz RA, Dusick AM, Vohr BR, Wright LL, Wrage LA, Poole WK. Growth in the neonatal intensive care unit influences neurodevelopmental and growth outcomes of extremely low birth weight infants. *Pediatrics*. 2006;117(4):1253-1261.

80. Wadhawan R, Oh W, Hintz SR, Blakely ML, Das A, Bell EF, et al. Neurodevelopmental outcomes of extremely low birth weight infants with spontaneous intestinal perforation or surgical necrotizing enterocolitis. *Journal of perinatology : official journal of the California Perinatal Association*. 2014;34(1):64-70.

81. Tsai AJ, Lasky RE, John SD, Evans PW, Kennedy KA. Predictors of neurodevelopmental outcomes in preterm infants with intraparenchymal hemorrhage. *Journal of perinatology : official journal of the California Perinatal Association*. 2014;34(5):399-404.

82. Merhar SL, Tabangin ME, Meinzen-Derr J, Schibler KR. Grade and laterality of intraventricular haemorrhage to predict 18-22 month neurodevelopmental outcomes in extremely low birthweight infants. *Acta paediatrica (Oslo, Norway : 1992)*. 2012;101(4):414-418.

83. Payne AH, Hintz SR, Hibbs AM, Walsh MC, Vohr BR, Bann CM, et al. Neurodevelopmental outcomes of extremely low-gestational-age neonates with low-grade periventricular-intraventricular hemorrhage. *JAMA pediatrics*. 2013;167(5):451-459.

84. Hintz SR, Kendrick DE, Stoll BJ, Vohr BR, Fanaroff AA, Donovan EF, et al. Neurodevelopmental and growth outcomes of extremely low birth weight infants after necrotizing enterocolitis. *Pediatrics*. 2005;115(3):696-703.

85. Stoll BJ, Hansen NI, Adams-Chapman I, Fanaroff AA, Hintz SR, Vohr B, et al. Neurodevelopmental and growth impairment among extremely low-birth-weight infants with neonatal infection. *Jama*. 2004;292(19):2357-2365.

86. Vohr BR, Wright LL, Dusick AM, Mele L, Verter J, Steichen JJ, et al. Neurodevelopmental and functional outcomes of extremely low birth weight infants in the National Institute of Child Health and Human Development Neonatal Research Network, 1993-1994. *Pediatrics*. 2000;105(6):1216-1226.

87. Lainwala S, Perritt R, Poole K, Vohr B. Neurodevelopmental and growth outcomes of extremely low birth weight infants who are transferred from neonatal intensive care units to level I or II nurseries. *Pediatrics*. 2007;119(5):e1079-1087.

88. Castro L, Yolton K, Haberman B, Roberto N, Hansen NI, Ambalavanan N, et al. Bias in reported neurodevelopmental outcomes among extremely low birth weight survivors. *Pediatrics*. 2004;114(2):404-410.

89. Hintz SR, Kendrick DE, Vohr BR, Kenneth Poole W, Higgins RD. Gender differences in neurodevelopmental outcomes among extremely preterm, extremely-low-birthweight infants. *Acta paediatrica (Oslo, Norway : 1992)*. 2006;95(10):1239-1248.

90. Hintz SR, Kendrick DE, Vohr BR, Poole WK, Higgins RD. Changes in neurodevelopmental outcomes at 18 to 22 months' corrected age among infants of less than 25 weeks' gestational age born in 1993-1999. *Pediatrics*. 2005;115(6):1645-1651.

91. Hintz SR, Kendrick DE, Wilson-Costello DE, Das A, Bell EF, Vohr BR, et al. Early-childhood neurodevelopmental outcomes are not improving for infants born at <25 weeks' gestational age. *Pediatrics*. 2011;127(1):62-70.

92. Laptook AR, O'Shea TM, Shankaran S, Bhaskar B. Adverse neurodevelopmental outcomes among extremely low birth weight infants with a normal head ultrasound: prevalence and antecedents. *Pediatrics*. 2005;115(3):673-680.

93. Messinger D, Lambert B, Bauer CR, Bann CM, Hamlin-Smith K, Das A. The Relationship between Behavior Ratings and Concurrent and Subsequent Mental and Motor Performance in Toddlers Born at Extremely Low Birth Weight. *Journal of early intervention*. 2010;32(3):214-233.

94. Shankaran S, Johnson Y, Langer JC, Vohr BR, Fanaroff AA, Wright LL, et al. Outcome of extremely-low-birth-weight infants at highest risk: gestational age < or =24 weeks, birth weight < or =750 g, and 1-minute Apgar < or =3. *Am J Obstet Gynecol*. 2004;191(4):1084-1091.

95. Vohr BR, Stephens BE, Higgins RD, Bann CM, Hintz SR, Das A, et al. Are outcomes of extremely preterm infants improving? Impact of Bayley assessment on outcomes. *The Journal of pediatrics*. 2012;161(2):222-228.e223.

96. Vohr BR, Tyson JE, Wright LL, Perritt RL, Li L, Poole WK. Maternal age, multiple birth, and extremely low birth weight infants. *The Journal of pediatrics*. 2009;154(4):498-503.e492.

97. Inhaled nitric oxide in term and near-term infants: neurodevelopmental follow-up of the neonatal inhaled nitric oxide study group (NINOS). *The Journal of pediatrics*. 2000;136(5):611-617.

98. Morriss FH, Jr., Saha S, Bell EF, Colaizy TT, Stoll BJ, Hintz SR, et al. Surgery and neurodevelopmental outcome of very low-birth-weight infants. *JAMA pediatrics*. 2014;168(8):746-754.

99. Wadhawan R, Oh W, Vohr BR, Wrage L, Das A, Bell EF, et al. Neurodevelopmental outcomes of triplets or higher-order extremely low birth weight infants. *Pediatrics*. 2011;127(3):e654-660.

100. Adams-Chapman I, Hansen NI, Stoll BJ, Higgins R. Neurodevelopmental outcome of extremely low birth weight infants with posthemorrhagic hydrocephalus requiring shunt insertion. *Pediatrics*. 2008;121(5):e1167-1177.

101. Dusick AM, Poindexter BB, Ehrenkranz RA, Lemons JA. Growth failure in the preterm infant: can we catch up? *Seminars in perinatology*. 2003;27(4):302-310.

102. Broitman E, Ambalavanan N, Higgins RD, Vohr BR, Das A, Bhaskar B, et al. Clinical data predict neurodevelopmental outcome better than head ultrasound in extremely low birth weight infants. *The Journal of pediatrics*. 2007;151(5):500-505, 505.e501-502.

103. Hintz SR, Van Meurs KP, Perritt R, Poole WK, Das A, Stevenson DK, et al. Neurodevelopmental outcomes of premature infants with severe respiratory failure enrolled in a randomized controlled trial of inhaled nitric oxide. *The Journal of pediatrics*. 2007;151(1):16-22, 22.e11-13.

104. Davis AS, Hintz SR, Van Meurs KP, Li L, Das A, Stoll BJ, et al. Seizures in extremely low birth weight infants are associated with adverse outcome. *The Journal of pediatrics*. 2010;157(5):720-725.e721-722.

105. Goldstein RF, Cotten CM, Shankaran S, Gantz MG, Poole WK. Influence of gestational age on death and neurodevelopmental outcome in premature infants with severe intracranial hemorrhage. *Journal of perinatology : official journal of the California Perinatal Association*. 2013;33(1):25-32.

106. Wadhawan R, Oh W, Vohr BR, Saha S, Das A, Bell EF, et al. Spontaneous intestinal perforation in extremely low birth weight infants: association with indometacin therapy and effects on neurodevelopmental outcomes at 18-22 months corrected age. *Archives of disease in childhood Fetal and neonatal edition*. 2013;98(2):F127-132.

107. Wadhawan R, Oh W, Perritt RL, McDonald SA, Das A, Poole WK, et al. Twin gestation and neurodevelopmental outcome in extremely low birth weight infants. *Pediatrics*. 2009;123(2):e220-227.

108. Mercier CE, Dunn MS, Ferrelli KR, Howard DB, Soll RF. Neurodevelopmental outcome of extremely low birth weight infants from the Vermont Oxford network: 1998-2003. *Neonatology*. 2010;97(4):329-338.

109. Petrini JR, Dias T, McCormick MC, Massolo ML, Green NS, Escobar GJ. Increased risk of adverse neurological development for late preterm infants. *The Journal of pediatrics*. 2009;154(2):169-176. e163.

110. Peralta-Carcelen M, Moses M, Adams-Chapman I, Gantz M, Vohr BR. Stability of neuromotor outcomes at 18 and 30 months of age after extremely low birth weight status. *Pediatrics*. 2009;123(5):e887-895.

111. Vohr BR, Msall ME, Wilson D, Wright LL, McDonald S, Poole WK. Spectrum of gross motor function in extremely low birth weight children with cerebral palsy at 18 months of age. *Pediatrics*. 2005;116(1):123-129.

112. Hack M, Horbar JD, Malloy MH, Tyson JE, Wright E, Wright L. Very low birth weight outcomes of the National Institute of Child Health and Human Development Neonatal Network. *Pediatrics*. 1991;87(5):587-597.

113. Horbar JD, Onstad L, Wright E. Predicting mortality risk for infants weighing 501 to 1500 grams at birth: a National Institutes of Health Neonatal Research Network report. *Critical care medicine*. 1993;21(1):12-18.

114. Fanaroff AA, Wright LL, Stevenson DK, Shankaran S, Donovan EF, Ehrenkranz RA, et al. Very-low-birth-weight outcomes of the National Institute of Child Health and Human Development Neonatal Research Network, May 1991 through December 1992. *Am J Obstet Gynecol*. 1995;173(5):1423-1431.

115. Hack M, Wright LL, Shankaran S, Tyson JE, Horbar JD, Bauer CR, et al. Very-low-birth-weight outcomes of the National Institute of Child Health and Human Development Neonatal Network, November 1989 to October 1990. *Am J Obstet Gynecol*. 1995;172(2 Pt 1):457-464.

116. Tyson JE, Younes N, Verter J, Wright LL. Viability, morbidity, and resource use among newborns of 501- to 800-g birth weight. National Institute of Child Health and Human Development Neonatal Research Network. *Jama*. 1996;276(20):1645-1651.

117. Horbar JD, Badger GJ, Lewit EM, Rogowski J, Shiono PH. Hospital and patient characteristics associated with variation in 28-day mortality rates for very low birth weight infants. Vermont Oxford Network. *Pediatrics*. 1997;99(2):149-156.

118. Stevenson DK, Wright LL, Lemons JA, Oh W, Korones SB, Papile LA, et al. Very low birth weight outcomes of the National Institute of Child Health and Human Development Neonatal Research Network, January 1993 through December 1994. *Am J Obstet Gynecol*. 1998;179(6 Pt 1):1632-1639.

119. Donovan EF, Ehrenkranz RA, Shankaran S, Stevenson DK, Wright LL, Younes N, et al. Outcomes of very low birth weight twins cared for in the National Institute of Child Health and Human Development Neonatal Research Network's intensive care units. *Am J Obstet Gynecol*. 1998;179(3 Pt 1):742-749.

120. Stevenson DK, Verter J, Fanaroff AA, Oh W, Ehrenkranz RA, Shankaran S, et al. Sex differences in outcomes of very low birthweight infants: the newborn male disadvantage. *Archives of disease in childhood Fetal and neonatal edition*. 2000;83(3):F182-185.

121. Lemons JA, Bauer CR, Oh W, Korones SB, Papile LA, Stoll BJ, et al. Very low birth weight outcomes of the National Institute of Child health and human development neonatal research network, January 1995 through December 1996. NICHD Neonatal Research Network. *Pediatrics*. 2001;107(1):E1.

122. Horbar JD, Badger GJ, Carpenter JH, Fanaroff AA, Kilpatrick S, LaCorte M, et al. Trends in mortality and morbidity for very low birth weight infants, 1991-1999. *Pediatrics*. 2002;110(1 Pt 1):143-151.

123. Shankaran S, Fanaroff AA, Wright LL, Stevenson DK, Donovan EF, Ehrenkranz RA, et al. Risk factors for early death among extremely low-birth-weight infants. *Am J Obstet Gynecol*. 2002;186(4):796-802.

124. Vohr BR, Wright LL, Dusick AM, Perritt R, Poole WK, Tyson JE, et al. Center differences and outcomes of extremely low birth weight infants. *Pediatrics*. 2004;113(4):781-789.

125. Rogowski JA, Horbar JD, Staiger DO, Kenny M, Carpenter J, Geppert J. Indirect vs direct hospital quality indicators for very low-birth-weight infants. *Jama*. 2004;291(2):202-209.

126. Lucey JF, Rowan CA, Shiono P, Wilkinson AR, Kilpatrick S, Payne NR, et al. Fetal infants: the fate of 4172 infants with birth weights of 401 to 500 grams--the Vermont Oxford Network experience (1996-2000). *Pediatrics*. 2004;113(6):1559-1566.

127. Hintz SR, Poole WK, Wright LL, Fanaroff AA, Kendrick DE, Laptook AR, et al. Changes in mortality and morbidities among infants born at less than 25 weeks during the post-surfactant era. *Archives of disease in childhood Fetal and neonatal edition*. 2005;90(2):F128-133.

128. Ambalavanan N, Carlo WA, Bobashev G, Mathias E, Liu B, Poole K, et al. Prediction of death for extremely low birth weight neonates. *Pediatrics*. 2005;116(6):1367-1373.

129. Morales LS, Staiger D, Horbar JD, Carpenter J, Kenny M, Geppert J, et al. Mortality among very low-birthweight infants in hospitals serving minority populations. *Am J Public Health*. 2005;95(12):2206-2212.

130. Ambalavanan N, Baibergenova A, Carlo WA, Saigal S, Schmidt B, Thorpe KE. Early prediction of poor outcome in extremely low birth weight infants by classification tree analysis. *The Journal of pediatrics*. 2006;148(4):438-444.

131. Fanaroff AA, Stoll BJ, Wright LL, Carlo WA, Ehrenkranz RA, Stark AR, et al. Trends in neonatal morbidity and mortality for very low birthweight infants. *Am J Obstet Gynecol*. 2007;196(2):147.e141-148.

132. Wadhawan R, Oh W, Perritt R, Laptook AR, Poole K, Wright LL, et al. Association between early postnatal weight loss and death or BPD in small and appropriate for gestational age extremely low-birth-weight infants. *Journal of perinatology : official journal of the California Perinatal Association*. 2007;27(6):359-364.

133. Bakewell-Sachs S, Medoff-Cooper B, Escobar GJ, Silber JH, Lorch SA. Infant functional status: the timing of physiologic maturation of premature infants. *Pediatrics*. 2009;123(5):e878-e886.

134. Gargus RA, Vohr BR, Tyson JE, High P, Higgins RD, Wrage LA, et al. Unimpaired outcomes for extremely low birth weight infants at 18 to 22 months. *Pediatrics*. 2009;124(1):112-121.

135. Chedid F, Shanteer S, Haddad H, Musharraf I, Shihab Z, Imran A, et al. Short-term outcome of very low birth weight infants in a developing country: comparison with the Vermont Oxford Network. *Journal of tropical pediatrics*. 2009;55(1):15-19.

136. De Nisi G, Berti M, Malossi R, Pederzini F, Pedrotti A, Valente A. Comparison of neonatal intensive care: Trento area versus Vermont Oxford Network. *Italian journal of pediatrics*. 2009;35(1):5.

137. Stoll BJ, Hansen NI, Bell EF, Shankaran S, Laptook AR, Walsh MC, et al. Neonatal outcomes of extremely preterm infants from the NICHD Neonatal Research Network. *Pediatrics*. 2010;126(3):443-456.

138. Bell EF, Hansen NI, Morriss FH, Jr., Stoll BJ, Ambalavanan N, Gould JB, et al. Impact of timing of birth and resident duty-hour restrictions on outcomes for small preterm infants. *Pediatrics*. 2010;126(2):222-231.

139. Murphy BP, Armstrong K, Ryan CA, Jenkins JG. Benchmarking care for very low birthweight infants in Ireland and Northern Ireland. *Archives of disease in childhood Fetal and neonatal edition*. 2010;95(1):F30-35.

140. Morrow AL, Meinzen-Derr J, Huang P, Schibler KR, Cahill T, Keddache M, et al. Fucosyltransferase 2 non-secretor and low secretor status predicts severe outcomes in premature infants. *The Journal of pediatrics*. 2011;158(5):745-751.

141. Rahman S, Salameh K, Al-Rifai H, Masoud A, Lutfi S, Salama H, et al. Gestational age specific neonatal survival in the State of Qatar (2003-2008) - a comparative study with international benchmarks. *Journal of the College of Physicians and Surgeons--Pakistan : JCPSP*. 2011;21(9):542-547.

142. De Jesus LC, Pappas A, Shankaran S, Kendrick D, Das A, Higgins RD, et al. Risk factors for post-neonatal intensive care unit discharge mortality among extremely low birth weight infants. *The Journal of pediatrics*. 2012;161(1):70-74.e71-72.

143. Ambalavanan N, Carlo WA, Tyson JE, Langer JC, Walsh MC, Parikh NA, et al. Outcome trajectories in extremely preterm infants. *Pediatrics*. 2012;130(1):e115-125.

144. Horbar JD, Carpenter JH, Badger GJ, Kenny MJ, Soll RF, Morrow KA, et al. Mortality and neonatal morbidity among infants 501 to 1500 grams from 2000 to 2009. *Pediatrics*. 2012;129(6):1019-1026.

145. Alleman BW, Bell EF, Li L, Dagle JM, Smith PB, Ambalavanan N, et al. Individual and center-level factors affecting mortality among extremely low birth weight infants. *Pediatrics*. 2013;132(1):e175-184.

146. Kumar P, Shankaran S, Ambalavanan N, Kendrick DE, Pappas A, Vohr BR, et al. Characteristics of extremely low-birth-weight infant survivors with unimpaired outcomes at 30 months of age. *Journal of perinatology : official journal of the California Perinatal Association*. 2013;33(10):800-805.

147. Randolph DA, Nolen TL, Ambalavanan N, Carlo WA, Peralta-Carcelen M, Das A, et al. Outcomes of extremely low birthweight infants with acidosis at birth. *Archives of disease in childhood Fetal and neonatal edition*. 2014;99(4):F263-268.

148. Davis AS, Hintz SR, Goldstein RF, Ambalavanan N, Bann CM, Stoll BJ, et al. Outcomes of extremely preterm infants following severe intracranial hemorrhage. *Journal of perinatology : official journal of the California Perinatal Association*. 2014;34(3):203-208.

149. Peralta-Carcelen M, Bailey K, Rector R, Gantz M. Behavioral and socioemotional competence problems of extremely low birth weight children. *Journal of perinatology : official journal of the California Perinatal Association*. 2013;33(11):887-892.

150. Bernstein IM, Horbar JD, Badger GJ, Ohlsson A, Golan A. Morbidity and mortality among very-low-birth-weight neonates with intrauterine growth restriction. The Vermont Oxford Network. *Am J Obstet Gynecol*. 2000;182(1 Pt 1):198-206.

151. Shankaran S, Laptook A, Wright LL, Ehrenkranz RA, Donovan EF, Fanaroff AA, et al. Whole-body hypothermia for neonatal encephalopathy: animal observations as a basis for a randomized, controlled pilot study in term infants. *Pediatrics*. 2002;110(2 Pt 1):377-385.

152. Shankaran S, Laptook AR, Ehrenkranz RA, Tyson JE, McDonald SA, Donovan EF, et al. Whole-body hypothermia for neonates with hypoxic-ischemic encephalopathy. *N Engl J Med*. 2005;353(15):1574-1584.

153. Ambalavanan N, Carlo WA, Shankaran S, Bann CM, Emrich SL, Higgins RD, et al. Predicting outcomes of neonates diagnosed with hypoxemic-ischemic encephalopathy. *Pediatrics*. 2006;118(5):2084-2093.

154. Laptook A, Tyson J, Shankaran S, McDonald S, Ehrenkranz R, Fanaroff A, et al. Elevated temperature after hypoxic-ischemic encephalopathy: risk factor for adverse outcomes. *Pediatrics*. 2008;122(3):491-499.

155. Oh W, Perritt R, Shankaran S, Merritts M, Donovan EF, Ehrenkranz RA, et al. Association between urinary lactate to creatinine ratio and neurodevelopmental outcome in term infants with hypoxic-ischemic encephalopathy. *The Journal of pediatrics*. 2008;153(3):375-378.

156. Shankaran S, Pappas A, Laptook AR, McDonald SA, Ehrenkranz RA, Tyson JE, et al. Outcomes of safety and effectiveness in a multicenter randomized, controlled trial of whole-body hypothermia for neonatal hypoxic-ischemic encephalopathy. *Pediatrics*. 2008;122(4):e791-798.

157. Mietzsch U, Parikh NA, Williams AL, Shankaran S, Lasky RE. Effects of hypoxic-ischemic encephalopathy and whole-body hypothermia on neonatal auditory function: a pilot study. *American journal of perinatology*. 2008;25(7):435-441.

158. Laptook AR, Shankaran S, Ambalavanan N, Carlo WA, McDonald SA, Higgins RD, et al. Outcome of term infants using apgar scores at 10 minutes following hypoxic-ischemic encephalopathy. *Pediatrics*. 2009;124(6):1619-1626.

159. Lasky RE, Parikh NA, Williams AL, Padhye NS, Shankaran S. Changes in the PQRST intervals and heart rate variability associated with rewarming in two newborns undergoing hypothermia therapy. *Neonatology*. 2009;96(2):93-95.

160. Parikh NA, Lasky RE, Garza CN, Bonfante-Mejia E, Shankaran S, Tyson JE. Volumetric and anatomical MRI for hypoxic-ischemic encephalopathy: relationship to hypothermia therapy and neurosensory impairments. *Journal of perinatology : official journal of the California Perinatal Association*. 2009;29(2):143-149.

161. Kwon JM, Guillet R, Shankaran S, Laptook AR, McDonald SA, Ehrenkranz RA, et al. Clinical seizures in neonatal hypoxic-ischemic encephalopathy have no independent impact on neurodevelopmental outcome: secondary analyses of data from the neonatal research network hypothermia trial. *Journal of child neurology*. 2011;26(3):322-328.

162. Pappas A, Shankaran S, Laptook AR, Langer JC, Bara R, Ehrenkranz RA, et al. Hypocarbia and adverse outcome in neonatal hypoxic-ischemic encephalopathy. *The Journal of pediatrics*. 2011;158(5):752-758.e751.

163. Shankaran S, Pappas A, McDonald SA, Laptook AR, Bara R, Ehrenkranz RA, et al. Predictive value of an early amplitude integrated electroencephalogram and neurologic examination. *Pediatrics*. 2011;128(1):e112-120.

164. Natarajan G, Pappas A, Shankaran S, Laptook AR, Walsh M, McDonald SA, et al. Effect of inborn vs. outborn delivery on neurodevelopmental outcomes in infants with hypoxic-ischemic encephalopathy: secondary analyses of the NICHD whole-body cooling trial. *Pediatr Res*. 2012;72(4):414-419.

165. Sant'Anna G, Laptook AR, Shankaran S, Bara R, McDonald SA, Higgins RD, et al. Phenobarbital and temperature profile during hypothermia for hypoxic-ischemic encephalopathy. *Journal of child neurology*. 2012;27(4):451-457.

166. Shankaran S, Barnes PD, Hintz SR, Laptook AR, Zaterka-Baxter KM, McDonald SA, et al. Brain injury following trial of hypothermia for neonatal hypoxic-ischaemic encephalopathy. *Archives of disease in childhood Fetal and neonatal edition*. 2012;97(6):F398-404.

167. Shankaran S, Laptook AR, McDonald SA, Higgins RD, Tyson JE, Ehrenkranz RA, et al. Temperature profile and outcomes of neonates undergoing whole body hypothermia for neonatal hypoxic-ischemic encephalopathy. *Pediatric critical care medicine : a journal of the Society of Critical Care Medicine and the World Federation of Pediatric Intensive and Critical Care Societies*. 2012;13(1):53-59.

168. Shankaran S, Laptook AR, Tyson JE, Ehrenkranz RA, Bann CM, Das A, et al. Evolution of encephalopathy during whole body hypothermia for neonatal hypoxic-ischemic encephalopathy. *The Journal of pediatrics*. 2012;160(4):567-572.e563.

169. Shankaran S, Pappas A, McDonald SA, Vohr BR, Hintz SR, Yolton K, et al. Childhood outcomes after hypothermia for neonatal encephalopathy. *N Engl J Med*. 2012;366(22):2085-2092.

170. Natarajan G, Shankaran S, Laptook AR, Pappas A, Bann CM, McDonald SA, et al. Apgar scores at 10 min and outcomes at 6-7 years following hypoxic-ischaemic encephalopathy. *Archives of disease in childhood Fetal and neonatal edition*. 2013;98(6):F473-479.

171. Vohr BR, Stephens BE, McDonald SA, Ehrenkranz RA, Laptook AR, Pappas A, et al. Cerebral palsy and growth failure at 6 to 7 years. *Pediatrics*. 2013;132(4):e905-914.

172. Laptook AR, McDonald SA, Shankaran S, Stephens BE, Vohr BR, Guillet R, et al. Elevated temperature and 6- to 7-year outcome of neonatal encephalopathy. *Annals of neurology*. 2013;73(4):520-528.

173. Cotten CM, Goldstein RF, McDonald SA, Goldberg RN, Salhab WA, Carlo WA, et al. Apolipoprotein E genotype and outcome in infants with hypoxic-ischemic encephalopathy. *Pediatr Res*. 2014;75(3):424-430.

174. Natarajan G, Shankaran S, Pappas A, Bann C, Tyson JE, McDonald S, et al. Functional status at 18 months of age as a predictor of childhood disability after neonatal hypoxic-ischemic encephalopathy. *Developmental medicine and child neurology*. 2014.

175. Fanaroff AA, Korones SB, Wright LL, Wright EC, Poland RL, Bauer CB, et al. A controlled trial of intravenous immune globulin to reduce nosocomial infections in very-low-birth-weight infants. National Institute of Child Health and Human Development Neonatal Research Network. *N Engl J Med*. 1994;330(16):1107-1113.

176. Stoll BJ, Hansen N, Fanaroff AA, Lemons JA. Enterobacter sakazakii is a rare cause of neonatal septicemia or meningitis in VLBW infants. *The Journal of pediatrics*. 2004;144(6):821-823.

177. Stoll BJ, Hansen N, Fanaroff AA, Wright LL, Carlo WA, Ehrenkranz RA, et al. To tap or not to tap: high likelihood of meningitis without sepsis among very low birth weight infants. *Pediatrics*. 2004;113(5):1181-1186.

178. Payne NR, Carpenter JH, Badger GJ, Horbar JD, Rogowski J. Marginal increase in cost and excess length of stay associated with nosocomial bloodstream infections in surviving very low birth weight infants. *Pediatrics*. 2004;114(2):348-355.

179. Cotten CM, McDonald S, Stoll B, Goldberg RN, Poole K, Benjamin DK, Jr. The association of third-generation cephalosporin use and invasive candidiasis in extremely low birth-weight infants. *Pediatrics*. 2006;118(2):717-722.

180. Benjamin DK, Stoll BJ, Fanaroff AA, McDonald SA, Oh W, Higgins RD, et al. Neonatal candidiasis among extremely low birth weight infants: risk factors, mortality rates, and neurodevelopmental outcomes at 18 to 22 months. *Pediatrics*. 2006;117(1):84-92.

181. Bassler D, Stoll BJ, Schmidt B, Asztalos EV, Roberts RS, Robertson CM, et al. Using a count of neonatal morbidities to predict poor outcome in extremely low birth weight infants: added role of neonatal infection. *Pediatrics*. 2009;123(1):313-318.

182. Benjamin DK, Jr., Stoll BJ, Gantz MG, Walsh MC, Sanchez PJ, Das A, et al. Neonatal candidiasis: epidemiology, risk factors, and clinical judgment. *Pediatrics*. 2010;126(4):e865-873.

183. Ang JY, Lua JL, Asmar BI, Shankaran S, Heyne RJ, Schelonka RL, et al. Nasopharyngeal carriage of Streptococcus pneumoniae in very low-birth-weight infants after administration of heptavalent pneumococcal conjugate vaccine. *Arch Pediatr Adolesc Med*. 2010;164(12):1173-1175.

184. Schelonka RL, Maheshwari A, Carlo WA, Taylor S, Hansen NI, Schendel DE, et al. T cell cytokines and the risk of blood stream infection in extremely low birth weight infants. *Cytokine*. 2011;53(2):249-255.

185. Greenberg RG, Benjamin DK, Jr., Gantz MG, Cotten CM, Stoll BJ, Walsh MC, et al. Empiric antifungal therapy and outcomes in extremely low birth weight infants with invasive candidiasis. *The Journal of pediatrics*. 2012;161(2):264-269.e262.

186. Wynn JL, Tan S, Gantz MG, Das A, Goldberg RN, Adams-Chapman I, et al. Outcomes following candiduria in extremely low birth weight infants. *Clinical infectious diseases : an official publication of the Infectious Diseases Society of America*. 2012;54(3):331-339.

187. Shane AL, Hansen NI, Stoll BJ, Bell EF, Sanchez PJ, Shankaran S, et al. Methicillin-resistant and susceptible Staphylococcus aureus bacteremia and meningitis in preterm infants. *Pediatrics*. 2012;129(4):e914-922.

188. Bliss JM, Wong AY, Bhak G, Laforce-Nesbitt SS, Taylor S, Tan S, et al. Candida virulence properties and adverse clinical outcomes in neonatal candidiasis. *The Journal of pediatrics*. 2012;161(3):441-447.e442.

189. Adams-Chapman I, Bann CM, Das A, Goldberg RN, Stoll BJ, Walsh MC, et al. Neurodevelopmental outcome of extremely low birth weight infants with Candida infection. *The Journal of pediatrics*. 2013;163(4):961-967.e963.

190. Fairlie T, Zell ER, Schrag S. Effectiveness of intrapartum antibiotic prophylaxis for prevention of early-onset group B streptococcal disease. *Obstetrics and gynecology*. 2013;121(3):570-577.

191. Donovan EF, Sparling K, Lake MR, Narendran V, Schibler K, Haberman B, et al. The investment case for preventing NICU-associated infections. *American journal of perinatology*. 2013;30(3):179-184.

192. Rogowski JA, Staiger D, Patrick T, Horbar J, Kenny M, Lake ET. Nurse staffing and NICU infection rates. *JAMA pediatrics*. 2013;167(5):444-450.

193. Uauy RD, Fanaroff AA, Korones SB, Phillips EA, Phillips JB, Wright LL. Necrotizing enterocolitis in very low birth weight infants: biodemographic and clinical correlates. National Institute of Child Health and Human Development Neonatal Research Network. *The Journal of pediatrics*. 1991;119(4):630-638.

194. Blakely ML, Lally KP, McDonald S, Brown RL, Barnhart DC, Ricketts RR, et al. Postoperative outcomes of extremely low birth-weight infants with necrotizing enterocolitis or isolated intestinal perforation: a prospective cohort study by the NICHD Neonatal Research Network. *Annals of surgery*. 2005;241(6):984-989; discussion 989-994.

195. Guillet R, Stoll BJ, Cotten CM, Gantz M, McDonald S, Poole WK, et al. Association of H2-blocker therapy and higher incidence of necrotizing enterocolitis in very low birth weight infants. *Pediatrics*. 2006;117(2):e137-142.

196. Blakely ML, Tyson JE, Lally KP, McDonald S, Stoll BJ, Stevenson DK, et al. Laparotomy versus peritoneal drainage for necrotizing enterocolitis or isolated intestinal perforation in extremely low birth weight infants: outcomes through 18 months adjusted age. *Pediatrics*. 2006;117(4):e680-687.

197. Pietz J, Achanti B, Lilien L, Stepka EC, Mehta SK. Prevention of necrotizing enterocolitis in preterm infants: a 20-year experience. *Pediatrics*. 2007;119(1):e164-170.

198. Cole CR, Hansen NI, Higgins RD, Ziegler TR, Stoll BJ. Very low birth weight preterm infants with surgical short bowel syndrome: incidence, morbidity and mortality, and growth outcomes at 18 to 22 months. *Pediatrics*. 2008;122(3):e573-582.

199. Cotten CM, Taylor S, Stoll B, Goldberg RN, Hansen NI, Sanchez PJ, et al. Prolonged duration of initial empirical antibiotic treatment is associated with increased rates of necrotizing enterocolitis and death for extremely low birth weight infants. *Pediatrics*. 2009;123(1):58-66.

200. Meinzen-Derr J, Morrow AL, Hornung RW, Donovan EF, Dietrich KN, Succop PA. Epidemiology of necrotizing enterocolitis temporal clustering in two neonatology practices. *The Journal of pediatrics*. 2009;154(5):656-661.

201. Fitzgibbons SC, Ching Y, Yu D, Carpenter J, Kenny M, Weldon C, et al. Mortality of necrotizing enterocolitis expressed by birth weight categories. *Journal of pediatric surgery*. 2009;44(6):1072-1075; discussion 1075-1076.

202. Singh R, Visintainer PF, Frantz ID, 3rd, Shah BL, Meyer KM, Favila SA, et al. Association of necrotizing enterocolitis with anemia and packed red blood cell transfusions in preterm infants. *Journal of perinatology : official journal of the California Perinatal Association*. 2011;31(3):176-182.

203. Cole CR, Hansen NI, Higgins RD, Bell EF, Shankaran S, Laptook AR, et al. Bloodstream infections in very low birth weight infants with intestinal failure. *The Journal of pediatrics*. 2012;160(1):54-59.e52.

204. Shah TA, Meinzen-Derr J, Gratton T, Steichen J, Donovan EF, Yolton K, et al. Hospital and neurodevelopmental outcomes of extremely low-birth-weight infants with necrotizing enterocolitis and spontaneous intestinal perforation. *Journal of perinatology : official journal of the California Perinatal Association*. 2012;32(7):552-558.

205. Hull MA, Fisher JG, Gutierrez IM, Jones BA, Kang KH, Kenny M, et al. Mortality and Management of Surgical Necrotizing Enterocolitis in Very Low Birth Weight Neonates: A Prospective Cohort Study. *Journal of the American College of Surgeons*. 2013.

206. Maheshwari A, Schelonka RL, Dimmitt RA, Carlo WA, Munoz-Hernandez B, Das A, et al. Cytokines associated with necrotizing enterocolitis in extremely-low-birth-weight infants. *Pediatr Res*. 2014;76(1):100-108.

207. Fisher JG, Jones BA, Gutierrez IM, Hull MA, Kang KH, Kenny M, et al. Mortality associated with laparotomy-confirmed neonatal spontaneous intestinal perforation: A prospective 5-year multicenter analysis. *Journal of pediatric surgery*. 2014;49(8):1215-1219.

208. Stoll BJ, Gordon T, Korones SB, Shankaran S, Tyson JE, Bauer CR, et al. Early-onset sepsis in very low birth weight neonates: a report from the National Institute of Child Health and Human Development Neonatal Research Network. *The Journal of pediatrics*. 1996;129(1):72-80.

209. Stoll BJ, Gordon T, Korones SB, Shankaran S, Tyson JE, Bauer CR, et al. Late-onset sepsis in very low birth weight neonates: a report from the National Institute of Child Health and Human Development Neonatal Research Network. *The Journal of pediatrics*. 1996;129(1):63-71.

210. Fanaroff AA, Korones SB, Wright LL, Verter J, Poland RL, Bauer CR, et al. Incidence, presenting features, risk factors and significance of late onset septicemia in very low birth weight infants. The National Institute of Child Health and Human Development Neonatal Research Network. *Pediatr Infect Dis J*. 1998;17(7):593-598.

211. Escobar GJ, Li D-k, Armstrong MA, Gardner MN, Folck BF, Verdi JE, et al. Neonatal sepsis workups in infants≥ 2000 grams at birth: a population-based study. *Pediatrics*. 2000;106(2):256-263.

212. Stoll BJ, Hansen N, Fanaroff AA, Wright LL, Carlo WA, Ehrenkranz RA, et al. Changes in pathogens causing early-onset sepsis in very-low-birth-weight infants. *N Engl J Med*. 2002;347(4):240-247.

213. Stoll BJ, Hansen N, Fanaroff AA, Wright LL, Carlo WA, Ehrenkranz RA, et al. Late-onset sepsis in very low birth weight neonates: the experience of the NICHD Neonatal Research Network. *Pediatrics*. 2002;110(2 Pt 1):285-291.

214. Edwards WH, Conner JM, Soll RF. The effect of prophylactic ointment therapy on nosocomial sepsis rates and skin integrity in infants with birth weights of 501 to 1000 g. *Pediatrics*. 2004;113(5):1195-1203.

215. Stoll BJ, Hansen NI, Higgins RD, Fanaroff AA, Duara S, Goldberg R, et al. Very low birth weight preterm infants with early onset neonatal sepsis: the predominance of gram-negative infections continues in the National Institute of Child Health and Human Development Neonatal Research Network, 2002-2003. *Pediatr Infect Dis J*. 2005;24(7):635-639.

216. Stoll BJ, Hansen NI, Sanchez PJ, Faix RG, Poindexter BB, Van Meurs KP, et al. Early onset neonatal sepsis: the burden of group B Streptococcal and E. coli disease continues. *Pediatrics*. 2011;127(5):817-826.

217. Weston EJ, Pondo T, Lewis MM, Martell-Cleary P, Morin C, Jewell B, et al. The burden of invasive early-onset neonatal sepsis in the United States, 2005-2008. *Pediatr Infect Dis J*. 2011;30(11):937-941.

218. Sood BG, Shankaran S, Schelonka RL, Saha S, Benjamin DK, Jr., Sanchez PJ, et al. Cytokine profiles of preterm neonates with fungal and bacterial sepsis. *Pediatr Res*. 2012;72(2):212-220.

219. Boghossian NS, Page GP, Bell EF, Stoll BJ, Murray JC, Cotten CM, et al. Late-onset sepsis in very low birth weight infants from singleton and multiple-gestation births. *The Journal of pediatrics*. 2013;162(6):1120-1124, 1124.e1121.

220. Wynn JL, Hansen NI, Das A, Cotten CM, Goldberg RN, Sanchez PJ, et al. Early sepsis does not increase the risk of late sepsis in very low birth weight neonates. *The Journal of pediatrics*. 2013;162(5):942-948.e941-943.

221. Escobar GJ, Puopolo KM, Wi S, Turk BJ, Kuzniewicz MW, Walsh EM, et al. Stratification of Risk of Early-Onset Sepsis in Newborns≥ 34 Weeks’ Gestation. *Pediatrics*. 2014;133(1):30-36.

222. Wright LL, Verter J, Younes N, Stevenson D, Fanaroff AA, Shankaran S, et al. Antenatal corticosteroid administration and neonatal outcome in very low birth weight infants: the NICHD Neonatal Research Network. *Am J Obstet Gynecol*. 1995;173(1):269-274.

223. Shankaran S, Bauer CR, Bain R, Wright LL, Zachary J. Relationship between antenatal steroid administration and grades III and IV intracranial hemorrhage in low birth weight infants. The NICHD Neonatal Research Network. *Am J Obstet Gynecol*. 1995;173(1):305-312.

224. Horbar JD. Antenatal corticosteroid treatment and neonatal outcomes for infants 501 to 1500 gm in the Vermont-Oxford Trials Network. *Am J Obstet Gynecol*. 1995;173(1):275-281.

225. Wright LL, Horbar JD, Gunkel H, Verter J, Younes N, Andrews EB, et al. Evidence from multicenter networks on the current use and effectiveness of antenatal corticosteroids in low birth weight infants. *Am J Obstet Gynecol*. 1995;173(1):263-269.

226. Gardner MO, Papile LA, Wright LL. Antenatal corticosteroids in pregnancies complicated by preterm premature rupture of membranes. *Obstetrics and gynecology*. 1997;90(5):851-853.

227. Horbar JD. Increasing use of antenatal corticosteroid therapy between 1990 and 1993 in Vermont Oxford Network. *Journal of perinatology : official journal of the California Perinatal Association*. 1997;17(4):309-313.

228. Demarini S, Dollberg S, Hoath SB, Ho M, Donovan EF. Effects of antenatal corticosteroids on blood pressure in very low birth weight infants during the first 24 hours of life. *Journal of perinatology : official journal of the California Perinatal Association*. 1999;19(6 Pt 1):419-425.

229. Lee BH, Stoll BJ, McDonald SA, Higgins RD. Adverse neonatal outcomes associated with antenatal dexamethasone versus antenatal betamethasone. *Pediatrics*. 2006;117(5):1503-1510.

230. Lee BH, Stoll BJ, McDonald SA, Higgins RD. Neurodevelopmental outcomes of extremely low birth weight infants exposed prenatally to dexamethasone versus betamethasone. *Pediatrics*. 2008;121(2):289-296.

231. Carlo WA, McDonald SA, Fanaroff AA, Vohr BR, Stoll BJ, Ehrenkranz RA, et al. Association of antenatal corticosteroids with mortality and neurodevelopmental outcomes among infants born at 22 to 25 weeks' gestation. *Jama*. 2011;306(21):2348-2358.

232. Supplemental Therapeutic Oxygen for Prethreshold Retinopathy Of Prematurity (STOP-ROP), a randomized, controlled trial. I: primary outcomes. *Pediatrics*. 2000;105(2):295-310.

233. Blair BM, O'Halloran H S, Pauly TH, Stevens JL. Decreased incidence of retinopathy of prematurity, 1995-1997. *Journal of AAPOS : the official publication of the American Association for Pediatric Ophthalmology and Strabismus / American Association for Pediatric Ophthalmology and Strabismus*. 2001;5(2):118-122.

234. Chow LC, Wright KW, Sola A. Can changes in clinical practice decrease the incidence of severe retinopathy of prematurity in very low birth weight infants? *Pediatrics*. 2003;111(2):339-345.

235. Bhola R, Purkiss T, Hunter S, Stewart D, Rychwalski PJ. Effect of granulocyte colony-stimulating factor on the incidence of threshold retinopathy of prematurity. *Journal of AAPOS : the official publication of the American Association for Pediatric Ophthalmology and Strabismus / American Association for Pediatric Ophthalmology and Strabismus*. 2009;13(5):450-453.

236. Sood BG, Madan A, Saha S, Schendel D, Thorsen P, Skogstrand K, et al. Perinatal systemic inflammatory response syndrome and retinopathy of prematurity. *Pediatr Res*. 2010;67(4):394-400.

237. Carlo WA, Higgins RD. Optimum oxygen therapy to prevent retinopathy of prematurity. *Expert review of ophthalmology*. 2010;5(5):583-585.

238. Carlo WA, Finer NN, Walsh MC, Rich W, Gantz MG, Laptook AR, et al. Target ranges of oxygen saturation in extremely preterm infants. *N Engl J Med*. 2010;362(21):1959-1969.

239. Kennedy KA, Wrage LA, Higgins RD, Finer NN, Carlo WA, Walsh MC, et al. Evaluating retinopathy of prematurity screening guidelines for 24- to 27-week gestational age infants. *Journal of perinatology : official journal of the California Perinatal Association*. 2014;34(4):311-318.

240. Atkinson LR, Escobar GJ, Takayama JI, Newman TB. Phototherapy use in jaundiced newborns in a large managed care organization: do clinicians adhere to the guideline? *Pediatrics*. 2003;111(5):e555-e561.

241. Bender GJ, Cashore WJ, Oh W. Ontogeny of bilirubin-binding capacity and the effect of clinical status in premature infants born at less than 1300 grams. *Pediatrics*. 2007;120(5):1067-1073.

242. Kuzniewicz MW, Escobar GJ, Newman TB. Impact of universal bilirubin screening on severe hyperbilirubinemia and phototherapy use. *Pediatrics*. 2009;124(4):1031-1039.

243. Hintz SR, Stevenson DK, Yao Q, Wong RJ, Das A, Van Meurs KP, et al. Is phototherapy exposure associated with better or worse outcomes in 501- to 1000-g-birth-weight infants? *Acta paediatrica (Oslo, Norway : 1992)*. 2011;100(7):960-965.

244. Morris BH, Oh W, Tyson JE, Stevenson DK, Phelps DL, O'Shea TM, et al. Aggressive vs. conservative phototherapy for infants with extremely low birth weight. *N Engl J Med*. 2008;359(18):1885-1896.

245. Tyson JE, Pedroza C, Langer J, Green C, Morris B, Stevenson D, et al. Does aggressive phototherapy increase mortality while decreasing profound impairment among the smallest and sickest newborns? *Journal of perinatology : official journal of the California Perinatal Association*. 2012;32(9):677-684.

246. Lasky RE, Church MW, Orlando MS, Morris BH, Parikh NA, Tyson JE, et al. The effects of aggressive vs. conservative phototherapy on the brainstem auditory evoked responses of extremely-low-birth-weight infants. *Pediatr Res*. 2012;71(1):77-84.

247. Morris BH, Tyson JE, Stevenson DK, Oh W, Phelps DL, O'Shea TM, et al. Efficacy of phototherapy devices and outcomes among extremely low birth weight infants: multi-center observational study. *Journal of perinatology : official journal of the California Perinatal Association*. 2013;33(2):126-133.

248. Clyman RI, Saha S, Jobe A, Oh W. Indomethacin prophylaxis for preterm infants: the impact of 2 multicentered randomized controlled trials on clinical practice. *The Journal of pediatrics*. 2007;150(1):46-50.e42.

249. Alfaleh K, Smyth JA, Roberts RS, Solimano A, Asztalos EV, Schmidt B. Prevention and 18-month outcomes of serious pulmonary hemorrhage in extremely low birth weight infants: results from the trial of indomethacin prophylaxis in preterms. *Pediatrics*. 2008;121(2):e233-238.

250. Laptook AR, Salhab W, Bhaskar B. Admission temperature of low birth weight infants: predictors and associated morbidities. *Pediatrics*. 2007;119(3):e643-649.

251. Lorch SA, Srinivasan L, Escobar GJ. Epidemiology of apnea and bradycardia resolution in premature infants. *Pediatrics*. 2011;128(2):e366-e373.

252. Stephens BE, Bann CM, Watson VE, Sheinkopf SJ, Peralta-Carcelen M, Bodnar A, et al. Screening for autism spectrum disorders in extremely preterm infants. *Journal of developmental and behavioral pediatrics : JDBP*. 2012;33(7):535-541.

253. Kuzniewicz MW, Wi S, Qian Y, Walsh EM, Armstrong MA, Croen LA. Prevalence and Neonatal Factors Associated with Autism Spectrum Disorders in Preterm Infants. *The Journal of pediatrics*. 2014;164(1):20-25.

254. Shankaran S, Papile LA, Wright LL, Ehrenkranz RA, Mele L, Lemons JA, et al. The effect of antenatal phenobarbital therapy on neonatal intracranial hemorrhage in preterm infants. *N Engl J Med*. 1997;337(7):466-471.

255. McCain GC, Donovan EF, Gartside P. Preterm infant behavioral and heart rate responses to antenatal phenobarbital. *Research in nursing & health*. 1999;22(6):461-470.

256. Shankaran S, Papile LA, Wright LL, Ehrenkranz RA, Mele L, Lemons JA, et al. Neurodevelopmental outcome of premature infants after antenatal phenobarbital exposure. *Am J Obstet Gynecol*. 2002;187(1):171-177.

257. Suresh GK, Horbar JD, Kenny M, Carpenter JH. Major birth defects in very low birth weight infants in the Vermont Oxford Network. *The Journal of pediatrics*. 2001;139(3):366-373.

258. Adams-Chapman I, Hansen NI, Shankaran S, Bell EF, Boghossian NS, Murray JC, et al. Ten-year review of major birth defects in VLBW infants. *Pediatrics*. 2013;132(1):49-61.

259. Turcotte LM, Georgieff MK, Ross JA, Feusner JH, Tomlinson GE, Malogolowkin MH, et al. Neonatal medical exposures and characteristics of low birth weight hepatoblastoma cases: A report from the Children's Oncology Group. *Pediatric blood & cancer*. 2014;61(11):2018-2023.

260. Finer NN, Horbar JD, Carpenter JH. Cardiopulmonary resuscitation in the very low birth weight infant: the Vermont Oxford Network experience. *Pediatrics*. 1999;104(3 Pt 1):428-434.

261. Escobar GJ, Braveman PA, Ackerson L, Odouli R, Coleman-Phox K, Capra AM, et al. A randomized comparison of home visits and hospital-based group follow-up visits after early postpartum discharge. *Pediatrics*. 2001;108(3):719-727.

262. Pappas A, Kendrick DE, Shankaran S, Stoll BJ, Bell EF, Laptook AR, et al. Chorioamnionitis and Early Childhood Outcomes Among Extremely Low-Gestational-Age Neonates. *JAMA pediatrics*. 2014.

263. Hintz SR, Kendrick DE, Vohr BR, Poole WK, Higgins RD. Community supports after surviving extremely low-birth-weight, extremely preterm birth: special outpatient services in early childhood. *Arch Pediatr Adolesc Med*. 2008;162(8):748-755.

264. The Neonatal Inhaled Nitric Oxide Study Group. Inhaled Nitric Oxide and Hypoxic Respiratory Failure in Infants With Congenital Diaphragmatic Hernia. *Pediatrics*. 1997;99(6):838-845.

265. Oh W, Fanaroff AA, Carlo WA, Donovan EF, McDonald SA, Poole WK. Effects of delayed cord clamping in very-low-birth-weight infants. *Journal of perinatology : official journal of the California Perinatal Association*. 2011;31 Suppl 1:S68-71.

266. Malloy MH, Onstad L, Wright E. The effect of cesarean delivery on birth outcome in very low birth weight infants. National Institute of Child Health and Human Development Neonatal Research Network. *Obstetrics and gynecology*. 1991;77(4):498-503.

267. Wadhawan R, Vohr BR, Fanaroff AA, Perritt RL, Duara S, Stoll BJ, et al. Does labor influence neonatal and neurodevelopmental outcomes of extremely-low-birth-weight infants who are born by cesarean delivery? *Am J Obstet Gynecol*. 2003;189(2):501-506.

268. Stoll BJ, Temprosa M, Tyson JE, Papile LA, Wright LL, Bauer CR, et al. Dexamethasone therapy increases infection in very low birth weight infants. *Pediatrics*. 1999;104(5):e63.

269. Hintz SR, Bann CM, Ambalavanan N, Cotten CM, Das A, Higgins RD. Predicting time to hospital discharge for extremely preterm infants. *Pediatrics*. 2010;125(1):e146-154.

270. Greenwood C, Morrow AL, Lagomarcino AJ, Altaye M, Taft DH, Yu Z, et al. Early empiric antibiotic use in preterm infants is associated with lower bacterial diversity and higher relative abundance of Enterobacter. *The Journal of pediatrics*. 2014;165(1):23-29.

271. Ohls RK, Ehrenkranz RA, Wright LL, Lemons JA, Korones SB, Stoll BJ, et al. Effects of early erythropoietin therapy on the transfusion requirements of preterm infants below 1250 grams birth weight: a multicenter, randomized, controlled trial. *Pediatrics*. 2001;108(4):934-942.

272. Tyson JE, Parikh NA, Langer J, Green C, Higgins RD. Intensive care for extreme prematurity--moving beyond gestational age. *N Engl J Med*. 2008;358(16):1672-1681.

273. Stephens BE, Bann CM, Poole WK, Vohr BR. NEURODEVELOPMENTAL IMPAIRMENT: PREDICTORS OF ITS IMPACT ON THE FAMILIES OF EXTREMELY LOW BIRTH WEIGHT INFANTS AT 18 MONTHS. *Infant mental health journal*. 2008;29(6):570-587.

274. Malcolm WF, Gantz M, Martin RJ, Goldstein RF, Goldberg RN, Cotten CM. Use of medications for gastroesophageal reflux at discharge among extremely low birth weight infants. *Pediatrics*. 2008;121(1):22-27.

275. Boghossian NS, Hansen NI, Bell EF, Stoll BJ, Murray JC, Carey JC, et al. Mortality and morbidity of VLBW infants with trisomy 13 or trisomy 18. *Pediatrics*. 2014;133(2):226-235.

276. Boghossian NS, Hansen NI, Bell EF, Stoll BJ, Murray JC, Laptook AR, et al. Survival and morbidity outcomes for very low birth weight infants with Down syndrome. *Pediatrics*. 2010;126(6):1132-1140.

277. Boghossian NS, Horbar JD, Carpenter JH, Murray JC, Bell EF. Major chromosomal anomalies among very low birth weight infants in the Vermont Oxford Network. *The Journal of pediatrics*. 2012;160(5):774-780.e711.

278. Boghossian NS, Horbar JD, Murray JC, Carpenter JH. Anthropometric charts for infants with trisomies 21, 18, or 13 born between 22 weeks gestation and term: the VON charts. *American journal of medical genetics Part A*. 2012;158a(2):322-332.

279. Chee YY, Wong KY, Low L. Review of primary hypothyroidism in very low birthweight infants in a perinatal centre in Hong Kong. *Journal of paediatrics and child health*. 2011;47(11):824-831.

280. Klein NP, Massolo ML, Greene J, Dekker CL, Black S, Escobar GJ. Risk factors for developing apnea after immunization in the neonatal intensive care unit. *Pediatrics*. 2008;121(3):463-469.

281. Navar-Boggan A, Halsey N, Escobar G, Golden W, Klein N. Underimmunization at discharge from the neonatal intensive care unit. *Journal of Perinatology*. 2012;32(5):363-367.

282. Navar-Boggan A, Halsey N, Golden W, Escobar G, Massolo M, Klein N. Risk of fever and sepsis evaluations after routine immunizations in the neonatal intensive care unit. *Journal of Perinatology*. 2010;30(9):604-609.

283. Schmidt B, Davis P, Moddemann D, Ohlsson A, Roberts RS, Saigal S, et al. Long-term effects of indomethacin prophylaxis in extremely-low-birth-weight infants. *N Engl J Med*. 2001;344(26):1966-1972.

284. Doyle NM, Gardner MO, Wells L, Qualls C, Papile LA. Outcome of very low birth weight infants exposed to antenatal indomethacin for tocolysis. *Journal of perinatology : official journal of the California Perinatal Association*. 2005;25(5):336-340.

285. Schmidt B, Roberts RS, Fanaroff A, Davis P, Kirpalani HM, Nwaesei C, et al. Indomethacin prophylaxis, patent ductus arteriosus, and the risk of bronchopulmonary dysplasia: further analyses from the Trial of Indomethacin Prophylaxis in Preterms (TIPP). *The Journal of pediatrics*. 2006;148(6):730-734.

286. Mirza H, Oh W, Laptook A, Vohr B, Tucker R, Stonestreet BS. Indomethacin prophylaxis to prevent intraventricular hemorrhage: association between incidence and timing of drug administration. *The Journal of pediatrics*. 2013;163(3):706-710.e701.

287. Batton B, Li L, Newman NS, Das A, Watterberg KL, Yoder BA, et al. Use of antihypotensive therapies in extremely preterm infants. *Pediatrics*. 2013;131(6):e1865-1873.

288. Batton BJ, Li L, Newman NS, Das A, Watterberg KL, Yoder BA, et al. Feasibility study of early blood pressure management in extremely preterm infants. *The Journal of pediatrics*. 2012;161(1):65-69.e61.

289. Shankaran S, Bauer CR, Bain R, Wright LL, Zachary J. Prenatal and perinatal risk and protective factors for neonatal intracranial hemorrhage. National Institute of Child Health and Human Development Neonatal Research Network. *Arch Pediatr Adolesc Med*. 1996;150(5):491-497.

290. Singh R, Gorstein SV, Bednarek F, Chou JH, McGowan EC, Visintainer PF. A predictive model for SIVH risk in preterm infants and targeted indomethacin therapy for prevention. *Scientific reports*. 2013;3:2539.

291. Armstrong MA, Osejo VG, Lieberman L, Carpenter DM, Pantoja PM, Escobar GJ. Perinatal substance abuse intervention in obstetric clinics decreases adverse neonatal outcomes. *Journal of Perinatology*. 2003;23(1):3-9.

292. Goler N, Armstrong M, Taillac C, Osejo V. Substance abuse treatment linked with prenatal visits improves perinatal outcomes: a new standard. *Journal of Perinatology*. 2008;28(9):597-603.

293. Gunderson EP, Croen LA, Chiang V, Yoshida CK, Walton D, Go AS. Epidemiology of peripartum cardiomyopathy: incidence, predictors, and outcomes. *Obstetrics & Gynecology*. 2011;118(3):583-591.

294. Phelps DL, Ward RM, Williams RL, Watterberg KL, Laptook AR, Wrage LA, et al. Pharmacokinetics and safety of a single intravenous dose of myo-inositol in preterm infants of 23-29 wk. *Pediatr Res*. 2013;74(6):721-729.

295. Hintz SR, Gaylord TD, Oh W, Fanaroff AA, Mele L, Stevenson DK, et al. Serum bilirubin levels at 72 hours by selected characteristics in breastfed and formula-fed term infants delivered by cesarean section. *Acta paediatrica (Oslo, Norway : 1992)*. 2001;90(7):776-781.

296. Ehrenkranz RA, Das A, Wrage LA, Poindexter BB, Higgins RD, Stoll BJ, et al. Early nutrition mediates the influence of severity of illness on extremely LBW infants. *Pediatr Res*. 2011;69(6):522-529.

297. Vohr BR, Poindexter BB, Dusick AM, McKinley LT, Wright LL, Langer JC, et al. Beneficial effects of breast milk in the neonatal intensive care unit on the developmental outcome of extremely low birth weight infants at 18 months of age. *Pediatrics*. 2006;118(1):e115-123.

298. Vohr BR, Poindexter BB, Dusick AM, McKinley LT, Higgins RD, Langer JC, et al. Persistent beneficial effects of breast milk ingested in the neonatal intensive care unit on outcomes of extremely low birth weight infants at 30 months of age. *Pediatrics*. 2007;120(4):e953-959.

299. Heller CD, O'Shea M, Yao Q, Langer J, Ehrenkranz RA, Phelps DL, et al. Human milk intake and retinopathy of prematurity in extremely low birth weight infants. *Pediatrics*. 2007;120(1):1-9.

300. Meinzen-Derr J, Poindexter B, Wrage L, Morrow AL, Stoll B, Donovan EF. Role of human milk in extremely low birth weight infants' risk of necrotizing enterocolitis or death. *Journal of perinatology : official journal of the California Perinatal Association*. 2009;29(1):57-62.

301. Brownell EA, Lussier MM, Hagadorn JI, McGrath JM, Marinelli KA, Herson VC. Independent Predictors of Human Milk Receipt at Neonatal Intensive Care Uni Discharge. *American journal of perinatology*. 2013.

302. De Jesus LC, Pappas A, Shankaran S, Li L, Das A, Bell EF, et al. Outcomes of small for gestational age infants born at <27 weeks' gestation. *The Journal of pediatrics*. 2013;163(1):55-60.e51-53.

303. Natarajan G, Shankaran S, McDonald SA, Das A, Stoll BJ, Higgins RD, et al. Circulating beta chemokine and MMP 9 as markers of oxidative injury in extremely low birth weight infants. *Pediatr Res*. 2010;67(1):77-82.

304. Poindexter BB, Langer JC, Dusick AM, Ehrenkranz RA. Early provision of parenteral amino acids in extremely low birth weight infants: relation to growth and neurodevelopmental outcome. *The Journal of pediatrics*. 2006;148(3):300-305.

305. Poindexter BB, Ehrenkranz RA, Stoll BJ, Koch MA, Wright LL, Oh W, et al. Effect of parenteral glutamine supplementation on plasma amino acid concentrations in extremely low-birth-weight infants. *The American journal of clinical nutrition*. 2003;77(3):737-743.

306. Poindexter BB, Ehrenkranz RA, Stoll BJ, Wright LL, Poole WK, Oh W, et al. Parenteral glutamine supplementation does not reduce the risk of mortality or late-onset sepsis in extremely low birth weight infants. *Pediatrics*. 2004;113(5):1209-1215.

307. Shankaran S, Langer JC, Kazzi SN, Laptook AR, Walsh M. Cumulative index of exposure to hypocarbia and hyperoxia as risk factors for periventricular leukomalacia in low birth weight infants. *Pediatrics*. 2006;118(4):1654-1659.

308. Walsh-Sukys MC, Tyson JE, Wright LL, Bauer CR, Korones SB, Stevenson DK, et al. Persistent pulmonary hypertension of the newborn in the era before nitric oxide: practice variation and outcomes. *Pediatrics*. 2000;105(1 Pt 1):14-20.

309. Kabra NS, Schmidt B, Roberts RS, Doyle LW, Papile L, Fanaroff A. Neurosensory impairment after surgical closure of patent ductus arteriosus in extremely low birth weight infants: results from the Trial of Indomethacin Prophylaxis in Preterms. *The Journal of pediatrics*. 2007;150(3):229-234, 234.e221.

310. Vanhaesebrouck S, Zonnenberg I, Vandervoort P, Bruneel E, Van Hoestenberghe MR, Theyskens C. Conservative treatment for patent ductus arteriosus in the preterm. *Archives of disease in childhood Fetal and neonatal edition*. 2007;92(4):F244-247.

311. Madan JC, Kendrick D, Hagadorn JI, Frantz ID, 3rd. Patent ductus arteriosus therapy: impact on neonatal and 18-month outcome. *Pediatrics*. 2009;123(2):674-681.

312. Benjamin JR, Smith PB, Cotten CM, Jaggers J, Goldstein RF, Malcolm WF. Long-term morbidities associated with vocal cord paralysis after surgical closure of a patent ductus arteriosus in extremely low birth weight infants. *Journal of perinatology : official journal of the California Perinatal Association*. 2010;30(6):408-413.

313. Archer JM, Yeager SB, Kenny MJ, Soll RF, Horbar JD. Distribution of and mortality from serious congenital heart disease in very low birth weight infants. *Pediatrics*. 2011;127(2):293-299.

314. Pappas A, Shankaran S, Hansen NI, Bell EF, Stoll BJ, Laptook AR, et al. Outcome of extremely preterm infants (<1,000 g) with congenital heart defects from the National Institute of Child Health and Human Development Neonatal Research Network. *Pediatric cardiology*. 2012;33(8):1415-1426.

315. Natarajan G, Shankaran S, McDonald SA, Das A, Ehrenkranz RA, Goldberg RN, et al. Association between blood spot transforming growth factor-beta and patent ductus arteriosus in extremely low-birth weight infants. *Pediatric cardiology*. 2013;34(1):149-154.

316. Horbar JD, Rogowski J, Plsek PE, Delmore P, Edwards WH, Hocker J, et al. Collaborative quality improvement for neonatal intensive care. NIC/Q Project Investigators of the Vermont Oxford Network. *Pediatrics*. 2001;107(1):14-22.

317. Cotten CM, Oh W, McDonald S, Carlo W, Fanaroff AA, Duara S, et al. Prolonged hospital stay for extremely premature infants: risk factors, center differences, and the impact of mortality on selecting a best-performing center. *Journal of perinatology : official journal of the California Perinatal Association*. 2005;25(10):650-655.

318. McCormick MC, Escobar GJ, Zheng Z, Richardson DK. Factors influencing parental satisfaction with neonatal intensive care among the families of moderately premature infants. *Pediatrics*. 2008;121(6):1111-1118.

319. Binder S, Hill K, Meinzen-Derr J, Greenberg JM, Narendran V. Increasing VLBW deliveries at subspecialty perinatal centers via perinatal outreach. *Pediatrics*. 2011;127(3):487-493.

320. Smith PB, Ambalavanan N, Li L, Cotten CM, Laughon M, Walsh MC, et al. Approach to infants born at 22 to 24 weeks' gestation: relationship to outcomes of more-mature infants. *Pediatrics*. 2012;129(6):e1508-1516.

321. Lake ET, Staiger D, Horbar J, Cheung R, Kenny MJ, Patrick T, et al. Association between hospital recognition for nursing excellence and outcomes of very low-birth-weight infants. *Jama*. 2012;307(16):1709-1716.

322. Soll RF, Edwards EM, Badger GJ, Kenny MJ, Morrow KA, Buzas JS, et al. Obstetric and neonatal care practices for infants 501 to 1500 g from 2000 to 2009. *Pediatrics*. 2013;132(2):222-228.

323. Puch-Kapst K, Juran R, Stoever B, Wauer RR. Radiation exposure in 212 very low and extremely low birth weight infants. *Pediatrics*. 2009;124(6):1556-1564.

324. Escobar G, Greene J, Hulac P, Kincannon E, Bischoff K, Gardner M, et al. Rehospitalisation after birth hospitalisation: patterns among infants of all gestations. *Archives of disease in childhood*. 2005;90(2):125-131.

325. Morris BH, Gard CC, Kennedy K. Rehospitalization of extremely low birth weight (ELBW) infants: are there racial/ethnic disparities? *Journal of perinatology : official journal of the California Perinatal Association*. 2005;25(10):656-663.

326. Ambalavanan N, Carlo WA, McDonald SA, Yao Q, Das A, Higgins RD. Identification of extremely premature infants at high risk of rehospitalization. *Pediatrics*. 2011;128(5):e1216-1225.

327. Ambalavanan N, Carlo WA, McDonald SA, Das A, Schendel DE, Thorsen P, et al. Cytokines and posthemorrhagic ventricular dilation in premature infants. *American journal of perinatology*. 2012;29(9):731-740.

328. Dollberg S, Demarini S, Donovan EF, Hoath SB. Maturation of thermal capabilities in preterm infants. *American journal of perinatology*. 2000;17(1):47-51.

329. McCormick MC, Escobar GJ, Zheng Z, Richardson DK. Place of birth and variations in management of late preterm (“near-term”) infants. Seminars in perinatology; 2006: Elsevier. p. 44-47.

330. Escobar GJ, Joffe S, Gardner MN, Armstrong MA, Folck BF, Carpenter DM. Rehospitalization in the first two weeks after discharge from the neonatal intensive care unit. *Pediatrics*. 1999;104(1):e2-e2.

331. Joffe S, Escobar GJ, Black SB, Armstrong MA, Lieu TA. Rehospitalization for respiratory syncytial virus among premature infants. *Pediatrics*. 1999;104(4):894-899.

332. Wade K, Lorch S, Bakewell-Sachs S, Medoff-Cooper B, Silber J, Escobar G. Pediatric care for preterm infants after NICU discharge: high number of office visits and prescription medications. *Journal of Perinatology*. 2008;28(10):696-701.

333. Lorch SA, Baiocchi M, Silber JH, Even‐Shoshan O, Escobar GJ, Small DS. The role of outpatient facilities in explaining variations in risk‐adjusted readmission rates between hospitals. *Health services research*. 2010;45(1):24-41.

334. Ray KN, Escobar GJ, Lorch SA. Premature infants born to adolescent mothers: health care utilization after initial discharge. *Academic pediatrics*. 2010;10(5):302-308.

335. Kuzniewicz MW, Parker S-J, Schnake-Mahl A, Escobar GJ. Hospital Readmissions and Emergency Department Visits in Moderate Preterm, Late Preterm, and Early Term Infants. *Clinics in perinatology*. 2013;40(4):753-775.

336. D'Angio CT, Heyne RJ, O'Shea TM, Schelonka RL, Shankaran S, Duara S, et al. Heptavalent pneumococcal conjugate vaccine immunogenicity in very-low-birth-weight, premature infants. *Pediatr Infect Dis J*. 2010;29(7):600-606.

337. Wynn JL, Li L, Cotten CM, Phelps DL, Shankaran S, Goldberg RN, et al. Blood stream infection is associated with altered heptavalent pneumococcal conjugate vaccine immune responses in very low birth weight infants. *Journal of perinatology : official journal of the California Perinatal Association*. 2013;33(8):613-618.

338. D'Angio CT, Murray TE, Li L, Heyne RJ, O'Shea TM, Schelonka RL, et al. Immunogenicity of Haemophilus influenzae type b protein conjugate vaccines in very low birth weight infants. *Pediatr Infect Dis J*. 2013;32(12):1400-1402.

339. Tyson JE, Wright LL, Oh W, Kennedy KA, Mele L, Ehrenkranz RA, et al. Vitamin A supplementation for extremely-low-birth-weight infants. National Institute of Child Health and Human Development Neonatal Research Network. *N Engl J Med*. 1999;340(25):1962-1968.

340. Ambalavanan N, Tyson JE, Kennedy KA, Hansen NI, Vohr BR, Wright LL, et al. Vitamin A supplementation for extremely low birth weight infants: outcome at 18 to 22 months. *Pediatrics*. 2005;115(3):e249-254.

341. Londhe VA, Nolen TL, Das A, Higgins RD, Tyson JE, Oh W, et al. Vitamin A supplementation in extremely low-birth-weight infants: subgroup analysis in small-for-gestational-age infants. *American journal of perinatology*. 2013;30(9):771-780.

342. Bell EF, Hansen NI, Brion LP, Ehrenkranz RA, Kennedy KA, Walsh MC, et al. Serum Tocopherol Levels in Very Preterm Infants After a Single Dose of Vitamin E at Birth. *Pediatrics*. 2013;132(6):e1626-e1633.

343. Wyckoff MH, Salhab WA, Heyne RJ, Kendrick DE, Stoll BJ, Laptook AR. Outcome of extremely low birth weight infants who received delivery room cardiopulmonary resuscitation. *The Journal of pediatrics*. 2012;160(2):239-244.e232.
